# Machine learning prediction and explainability analysis of high strength glass powder concrete using SHAP PDP and ICE

**DOI:** 10.1038/s41598-025-04762-2

**Published:** 2025-07-01

**Authors:** Muhammad Sarmad Mahmood, Tariq Ali, Inamullah Inam, Muhammad Zeeshan Qureshi, Syed Salman Ahmad Zaidi, Muwaffaq Alqurashi, Hawreen Ahmed, Muhammad Adnan, Abdul Hakim Hotak

**Affiliations:** 1 Department of Civil Engineering, Swedish College of Engineering and Technology, Wah Cantt, 47080 Pakistan; 2 Department of Civil Engineering, Laghman University, Mehtarlam, Afghanistan; 3 Department of Civil Engineering, University of Engineering and Technology, Taxila, Pakistan; 4 Department of Civil Engineering, Wah Engineering College, University of Wah, Wah Cantt, 47040 Pakistan; 5 Department of Civil Engineering, College of Engineering, Taif University, P.O. Box 11099, Taif, 21944 Saudi Arabia; 6 Department of Highway and Bridge Engineering, Technical Engineering College, Erbil Polytechnic University, Erbil, 44001 Iraq; 7 School of Civil Engineering, Tianjin University, Tianjin, 300350 China

**Keywords:** High-strength concrete, Glass powder concrete, Machine learning, Hybrid optimization, SHAP analysis, Engineering, Materials science

## Abstract

Achieving high-strength concrete (HSC) with sustainable supplementary cementitious materials (SCMs) remains a significant challenge in the construction industry. Although glass powder has shown promise as a partial cement substitute, its specific impact on HSC growth is still unclear. This study aims to evaluate the compressive strength (CS) of high strength glass-powder concrete (HSGPC) using machine learning (ML) models and enhance predictive accuracy through hybrid optimization techniques. A dataset comprising 598 points was compiled, considering cement, glass powder, aggregates, water, superplasticizer, and curing days as key input parameters. Three standalone ML models—K-Nearest Neighbors (KNN), Random Forest (RF), and Extreme Gradient Boosting (XGB)—were trained, with RF achieving R² = 0.963 and XGB achieving R² = 0.946 on the test set. To further enhance performance, XGB was optimized using Particle Swarm Optimization (PSO), Firefly Algorithm (FA), and Grey Wolf Optimizer (GWO). Among these, XGB-GWO demonstrated the highest accuracy, with R² improving to 0.991 and MSE decreasing significantly from 83.95 to 14.42, resulting in an 82.82% error reduction. SHAP, PDP, and ICE analyses identified superplasticizer dosage, curing days, and coarse aggregate as the most influential parameters affecting compressive strength (CS). PDP and ICE validated these findings, showing reduced strength gains beyond 600 kg/m³ of cement and a decline beyond 800 kg/m³ of coarse aggregate. This study highlights the potential of ML-driven optimization for sustainable concrete design, offering an efficient, data-driven approach to optimizing material proportions for high-strength, eco-friendly concrete.

**Supplementary Information:**

The online version contains supplementary material available at 10.1038/s41598-025-04762-2.

## Introduction

In concrete, Ordinary Portland Cement (OPC) has been largely replaced by supplemental cementitious materials (SCMs). European cement manufacturers, for example, have replaced 25% of OPC clinkers during the past 20 years with alternative SCMs^1,2^. Fly ash caught the interest of researchers as a new and potential cementitious material because of its pozzolanic qualities and the existence of amorphous silica and alumina. According to recent research, replacing 40–60% of the calcium hydroxide (Ca(OH)_2_) generated during cement hydration, which results in the formation of more calcium silicate hydrate (C-S-H) and calcium aluminate hydrate (C-A-S-H) gel, can gradually increase the strength and durability of concrete^3–5^. Nonetheless, various studies indicate that the potential for substituting cement with elevated fly ash content may be constrained by the early strength development of concrete^6,7^.

The researchers utilized various methods to mitigate the initial reduction in CS of concrete comprising high volumes of fly ash, including the addition of lime to enhance the solubility of fly ash and accelerate the pozzolanic reaction, as well as making fly ash particles finer to increase their reactivity^6,8^. The utilization of industrial by-products, including blast furnace slag and silica fume, has gained popularity due to their substantial amorphous silica content, which enhances the early strength of such concrete^9^. Recycled waste glass, primarily soda-lime glass, is a non-biodegradable solid that has gained increased attention recently as a sustainable alternative to Portland cement in building materials. It has become widely recognized that pulverized glass debris from bottles may include significant amounts of silica and alumina, which seem to be non-crystalline (high amorphous silica concentration)^10,11^.

According to the preliminary work by Shao et al.^12^, glass powder finely crushed to less than 38 μm may exhibit pozzolanic activity. Due to the pozzolanic reaction of the glass powder, concrete with 30% glass powder as SCM exhibited reduced CS before 28 days but enhanced strength at 90 days. Moreover, like other SCMs, glass powder with long curing period^13,14^ and smaller particle size is more pozzolan^12,15^. Dyer and Dhir^16^ found that the leaching of alkalis from glass particles accelerated cement hydration when glass powder was utilized as a replacement. However, the amount of free alkalis is inadequate to compensate for the hydration and early-age strength reduction caused by cement dilution. Du and Tan^17^ as well as Schwarz et al.^18^ achieved similar findings. Moreover, when 10% glass powder was used in place of cement in concrete, Schwarz et al.^10^ examined the durability properties and found that there was a decrease in both water absorption and chloride penetration. However, at every testing interval up to 90 days, its CS was inferior to that of conventional cement concrete. Matos and Sousa-Coutinho^19^ studied the durability properties of masonry by substituting the 10% and 20% glass powder for cement. The replacement of glass powder decreased chloride diffusion without influencing water sorptivity or carbonation resistance. Carsana et al.^20^ reported that glass powder exhibited superior long-term pozzolanic activity compared to fly ash and natural pozzolans over a duration of seven years at a concentration of 30%. The literature indicates that glass powder can substitute a maximum of 30% of the cement component in mortar or concrete. Furthermore, glass powder has been utilized as SCM in concrete in substantial amounts (up to 60% by weight of OPC) in certain prior studies^16,21^. Such concrete was found to have both fresh and hardened characteristics. While the strength at 28 days increased, especially at 30% replacement, the strength at younger ages (less than 7 days) decreased steadily with the amount of glass powder. Additionally, the concrete was denser and more compacted due to the microstructure in the interfacial transition zone (ITZ). As a result, a significant increase in durability qualities has been noted.

It is evident that compressive strength (CS) is the key mechanical property. CS must be determined by compression tests carried out in compliance with applicable standards under particular curing times and ambient circumstances. All the constituents such as OPC, glass powder, water, fine and coarse aggregate show strong correlations with the CS of GPC^22,23^. Extensive experiments requiring a large investment of time, money, and resources are necessary if researchers hope to comprehend the interaction between CS and other elements. As a result, to represent the obtained data, researchers usually turn to using straightforward empirical formulae. However, these formulas are typically inadequate. This is because the correlations between variables and CS are complex and nonlinear and cannot be adequately captured by a single universal Eqs^24,25^.. Machine learning (ML) approaches are increasingly being used to predict the mechanical characteristics of materials due to artificial intelligence’s remarkable nonlinear fitting capacity^26–30^.

While a few studies have used ML techniques to predict the CS of glass powder concrete, most have been limited to normal-strength concrete and lack optimization techniques for improving predictive performance. Additionally, limited studies have explored the interpretability of these models through feature importance analysis. This study addresses these gaps by integrating three advanced metaheuristic algorithms, Particle Swarm Optimization (PSO), Firefly Algorithm (FA), and Grey Wolf Optimizer (GWO) to fine-tune the Extreme Gradient Boosting (XGB) model for accurate prediction of HSGPC compressive strength. In addition to benchmarking against baseline models (KNN, RF, and XGB), the study applies SHapley Additive Explanations (SHAP), Partial Dependence (PDP), and Individual Conditional Expectation (ICE) analyses to interpret feature effects. The study also employs Taylor diagrams for visual model comparison and robustness assessment. The key objectives include: (1) applying and comparing standalone ML models (KNN, RF, XGB); (2) improving performance via nature-inspired optimization techniques; and (3) conducting a comprehensive interpretability and statistical outlier analysis to ensure model reliability and engineering relevance.

The paper is systematically structured to ensure a clear progression of the research. Section “Research Significance”, elaborates on the research gap and the scope of the current study. Section “Research Methodology”, provides a detailed overview of data collection, dataset characteristics, and model development. Section “Results and Discussion”, presents the performance evaluation of various ML models along with an in-depth interpretability analysis. Section “Future Perspectives” outlines the future perspectives and research limitations, highlighting potential advancements and challenges. Finally, Section. “Conclusion”, summarizes the key findings and contributions of this study.

### Research significance

The application of ML algorithms to predict unseen data particularly in civil engineering with significant outcomes is gaining popularity. Predictions regarding the mechanical characteristics of concrete are prevalent, however acquiring requisite data using conventional approaches necessitates costly and difficult laboratory procedures.

Recent research has employed ML models to improve the predictive accuracy of concrete properties. In this regard, Neural networks, particularly backpropagation neural networks (BPNN), have successfully predicted the CS of ceramic waste slag concrete with notable accuracy^31^. Similarly, ensemble models like RF and boosting have shown promise. RF excelled in predicting various attributes of fly ash-included SCC, while boosting models significantly improved CS predictions using recycled coarse aggregates (RCA) and SCMs, emphasizing the critical impact of RCA replacement rates^32,33^. In one study conducted by Singh et al.^34^, the CS of high-performance concrete was predicted using ML models including MLP, SVR, and XGB, with XGB optimized by a genetic algorithm achieving the highest accuracy across multinational datasets. Another study by Parhi et al.^35^ demonstrated that using the Spotted Hyena Optimization algorithm to tune XGB hyperparameters significantly improved prediction accuracy of concrete strength over traditional grid and random search methods, with SHAP analysis highlighting concrete age as the most influential factor. Additionally, support vector machines (SVM) have been explored for their potential in predicting SCC concrete properties, though smaller sample sizes might constrain their effectiveness^36^. This body of work illustrates the effectiveness of advanced ML models in predicting concrete behaviors under varied conditions, including high temperatures where XGB outperformed other models^37^.

The utilization of glass powder as a replacement for OPC in concrete has been thoroughly investigated owing to its pozzolanic properties and sustainability advantages. Kovačević et al.^38^ developed multiple ML models, including Gaussian Process Regression (GPR), SVM, DT, and ensemble models such as RF and Boosted Trees, to forecast glass powder concrete’s CS. With an RMSE of 3.98 MPa and a R of 0.9244, their analysis showed that the GPR model with an ARD exponential covariance function had the best predicted accuracy. Gao and Ma^39^ compared the predictive capabilities of response surface methodology (RSM) and ANN for estimating CS in concrete containing waste glass powder and eggshell powder. ANN outperformed RSM, achieving an R² of 0.956 for CS prediction, demonstrating the superior learning capability of deep learning approaches in handling complex material interactions. Similarly, Nassar and Sohaib^40^ explored the use of ensemble learning, employing a stacking approach that combines AdaBoost, SVM, XGB, and DT models. Their results demonstrated that the stacking ensemble model had the greatest prediction accuracy, closely matching actual data, when used to forecast the 28-day CS of concrete containing 10–25% glass powder as a cement substitute. Xu et al.^22^ advanced ML-based predictions by integrating nature-inspired optimization algorithms with traditional models. They utilized four standalone ML models (BPNN, XGB, SVR, and RF), along with hybrid models optimized using the Sparrow Search Algorithm (SSA). The SSA-XGB model achieved the highest accuracy (R² = 0.9645, RMSE = 3.064 MPa), demonstrating the significant role of cement content and curing age in determining the strength of glass powder concrete. The study also performed a feature importance analysis using PDP to explore the impact of glass powder dosage, curing age, and cement content on CS. Table 1 provides a summary of previous studies, including references, employed ML models, dataset sizes, and interpretability analyses such as SHAP and PDP.

**Table 1:** Summary of previous Studies.

Reference	Concrete Type	SCM	No. of Inputs	Outputs	Dataset	Models employed	Analysis methods
^41^	High Strength	Fly Ash, GGBFS	8	CS	1030	ANN, bagging, boosting	-
^42^	Normal	Rice husk ash	6	CS	192	ADB, DT, bagging	Sensitivity analysis
^37^	Normal	-	9	CS	207	XGB, MLP, SVM	Sensitivity analysis
^38^	Normal	Glass powder	6	CS	70	GPR, SVM, DT, RF, Boosted Trees	-
^39^	Normal	Silica fume, eggshell powder, waste glass powder	7	CS	225	RSM, ANN	-
^40^	Normal	Waste glass	6	CS	N. A	Stacking (AdaBoost, SVM, XGB, DT)	-
^22^	Normal	Glass powder	6	CS	1045	BPNN, XGB, SVR, RF, SSA-XGB, SSA-BPNN, SSA-SVR, SSA-RFR	SHAP, feature importance, PDP
This Study	High Strength	Glass powder	7	CS	598	KNN, RF, XGB, XGB-PSO, XGB-FA, XGB-GWO	SHAP, feature importance, PDP, ICE

According to Table 1, the majority of research has been restricted to normal-strength concrete and has not concentrated on applying ML approaches to forecast the CS of glass powder concrete. Furthermore, extensive interpretability analyses such as PDP, ICE, and SHAP have not been comprehensively performed to evaluate the specific influence of GP and other input variables. To address this research gap, the present study employes advanced nature-inspired optimization algorithms, including PSO, FA, and GWO, to fine-tune the XGB model for accurately predicting the CS of HSGPC. Additionally, this study benchmarks the performance of the optimized XGB model against individual ML models, including DT, RF and XGB, thereby enhancing model generalization and prediction accuracy. Furthermore, the developed models are thoroughly analyzed using explainability analysis to ensure transparency in decision-making to facilitate practical applications.

## Research methodology

This study follows a structured approach to forecast the CS of HSGPC using ML models and optimization techniques. The overall methodology consists of four key stages.

- Data collection and preprocessing.
- Model development.
- Performance evaluation.
- Explainability analysis.

**Fig. 1:**
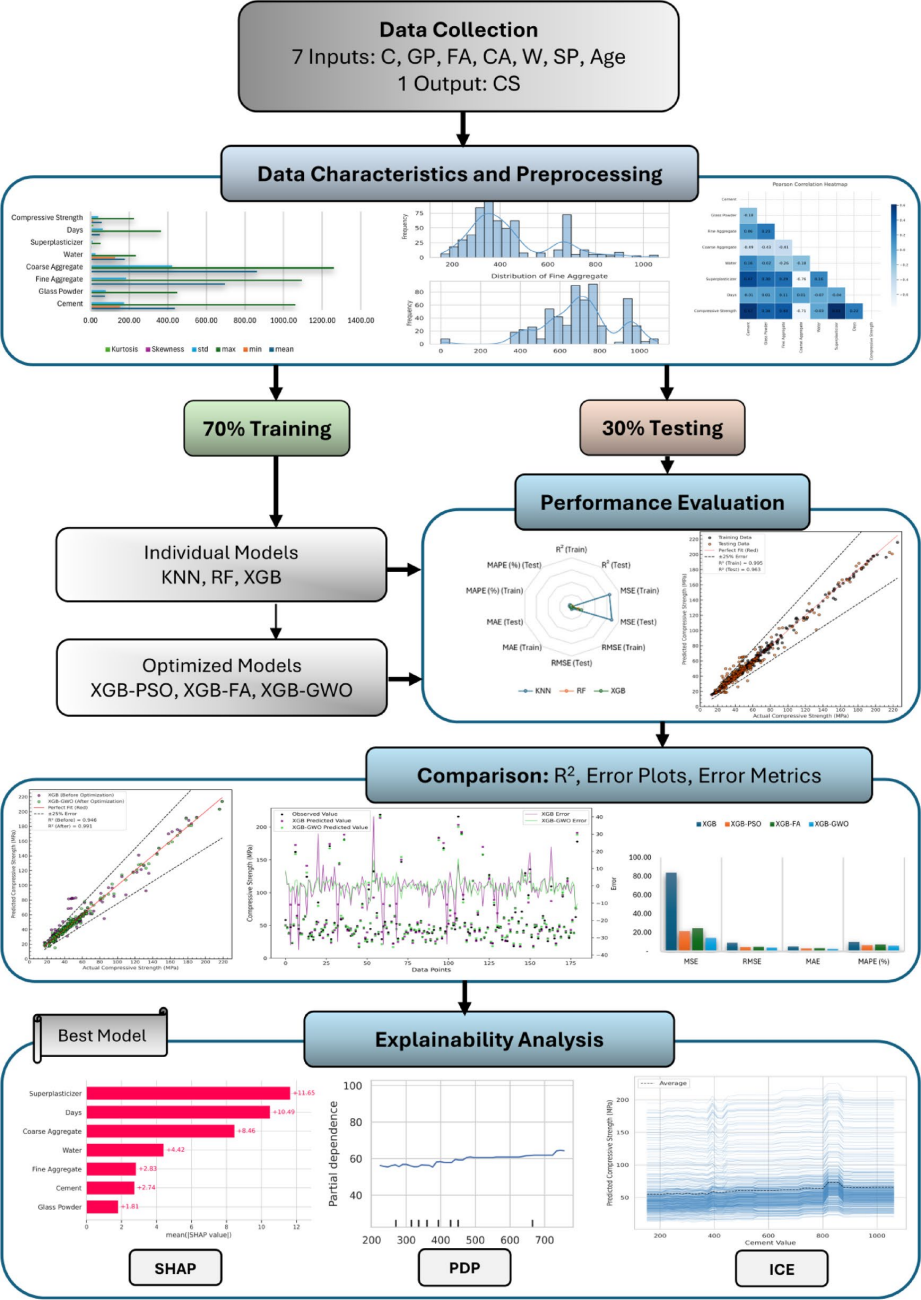
Research methodology framework.

The dataset, compiled from published experimental studies, undergoes preprocessing to ensure data quality. Three individual ML models—KNN, RF, and XGB—are trained and evaluated, followed by optimization of XGB using PSO, FA, and GWO. Performance is assessed through R², MSE, RMSE, MAE, and MAPE metrics, with a comparative analysis between models. Additionally, explainability analysis using SHAP, PDP, and ICE provides insights into feature influence and prediction trends. The overall research methodology is illustrated in Fig. 1.

### Dataset collection and characteristics

The dataset utilized in this investigation was collected from experimental studies on the mechanical characteristics of HSGPC that have already been published. The details of the sources and corresponding datasets (X) are presented in Table 2. Moreover, the complete dataset utilized in this study is attached as a **supplementary file (S1-S2).** The compiled dataset consists of 598 data points, each representing an experimental mix with specific material proportions and corresponding CS values.

**Table 2:** Dataset Sources.

Sr. #	Ref.	X	Sr. #	Ref.	X	Sr. #	Ref.	X
1.	^43^	12	2.	^44^	15	3.	^45^	08
4.	^46^	06	5.	^47^	15	6.	^1^	15
7.	^48^	15	8.	^49^	38	9.	^50^	20
10.	^51^	36	11.	^52^	30	12.	^53^	30
13.	^54^	30	14.	^55^	27	15.	^56^	24
16.	^57^	20	17.	^58^	30	18.	^17^	18
19.	^59^	18	20.	^60^	16	21.	^61^	72
22.	^62^	24	23.	^63^	33	24.	^64^	24
25.	^65^	16	26.	^66^	6	**Total**		**598**

The input parameters considered in this study include seven key mix design variables, which significantly influence the CS of HSGPC. These parameters are Cement Content ‘C’ (kg/m³), Glass Powder Content ‘GP’ (kg/m³), Fine Aggregate ‘FA’ (kg/m³), Coarse Aggregate ‘CA’ (kg/m³), Water Content ‘W’ (kg/m³), Superplasticizer Dosage ‘SP’ (kg/m³), Curing Age ‘D’ (days). The dependent variable in this study is the compressive strength ‘CS’ (MPa), measured at different curing ages. High-strength concrete (HSC) is generally defined as concrete with CS exceeding 40 MPa at 28 days. Such concrete exhibits superior mechanical properties, providing it appropriate for structural applications that demand increased durability, load-bearing capability, and sustainability. Table 3 provides a statistical overview of the dataset, highlighting key descriptive statistics such as mean, minimum, quartiles (25%, 50%, 75%), maximum, standard deviation, variance, skewness and kurtosis for each input variable and the output variable.

**Table 3:** Descriptive statistics of the dataset.

	C	GP	FA	CA	W	SP	D	CS
**Mean**	437.26	74.35	696.29	861.32	178.09	6.47	48.85	58.46
**Minimum**	152	0	0	0	127.5	0	1	13
**25%**	320	20	580	825	157	1.6	7	37.013
**50%**	384	61.5	702	1069.8	175	3.08	28	47
**75%**	474.5	107.2	786	1108	195.5	5.9	90	62.61
**Maximum**	1062	450	1094	1260	234	52.5	365	225
**Standard Deviation**	174.10	79.19	183.82	422.60	25.90	10.31	62.98	38.92
**Variance**	30311.9	6270.64	33788.3	178590.4	670.57	106.32	3966.96	1514.72
**Skewness**	1.15	1.86	−0.54	−1.44	0.41	2.65	3.40	2.15
**Kurtosis**	0.90	4.74	1.45	0.32	−0.93	6.70	13.92	4.42

**Fig. 2:**
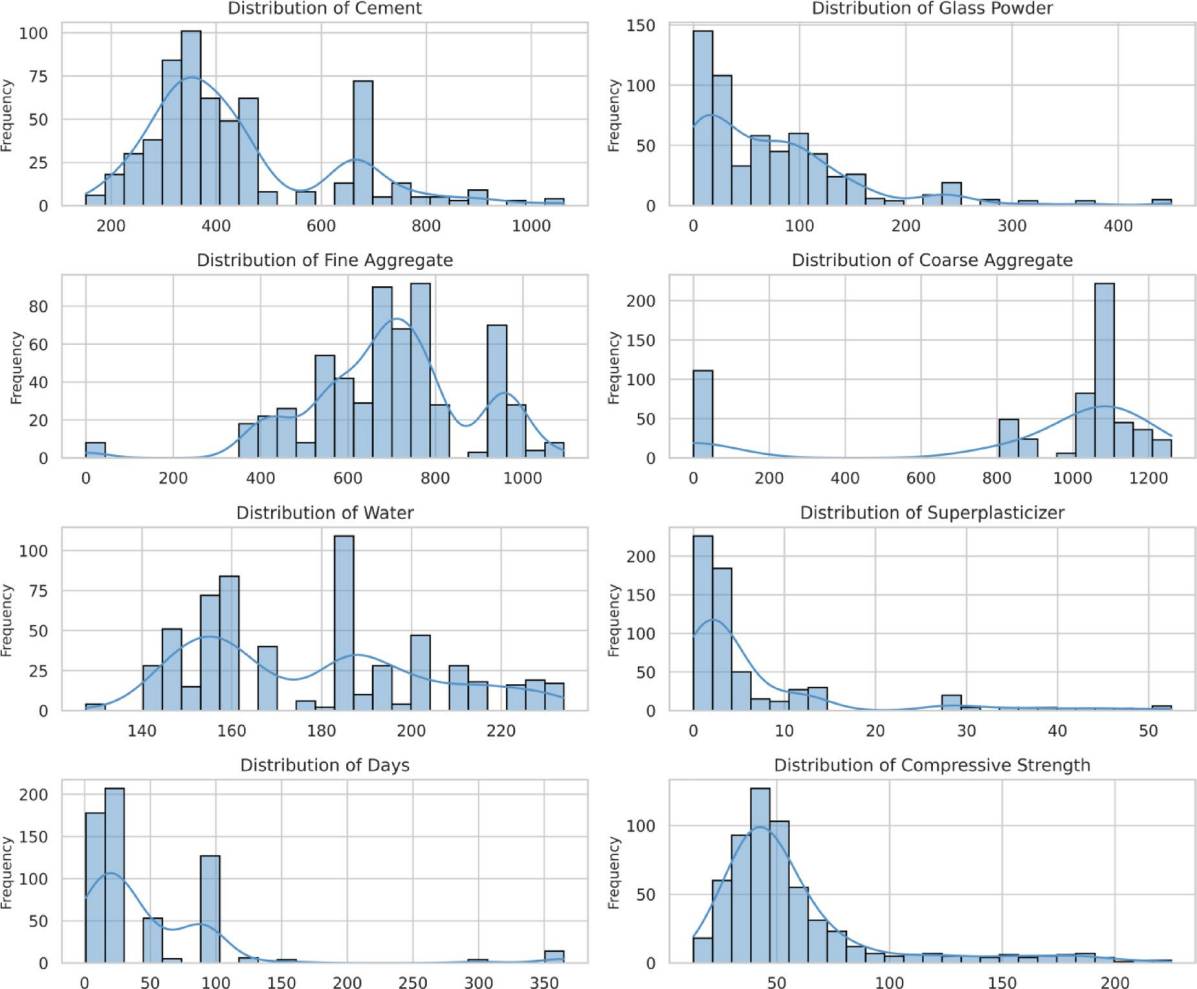
Histogram Distribution of Variables.

**Fig. 3:**
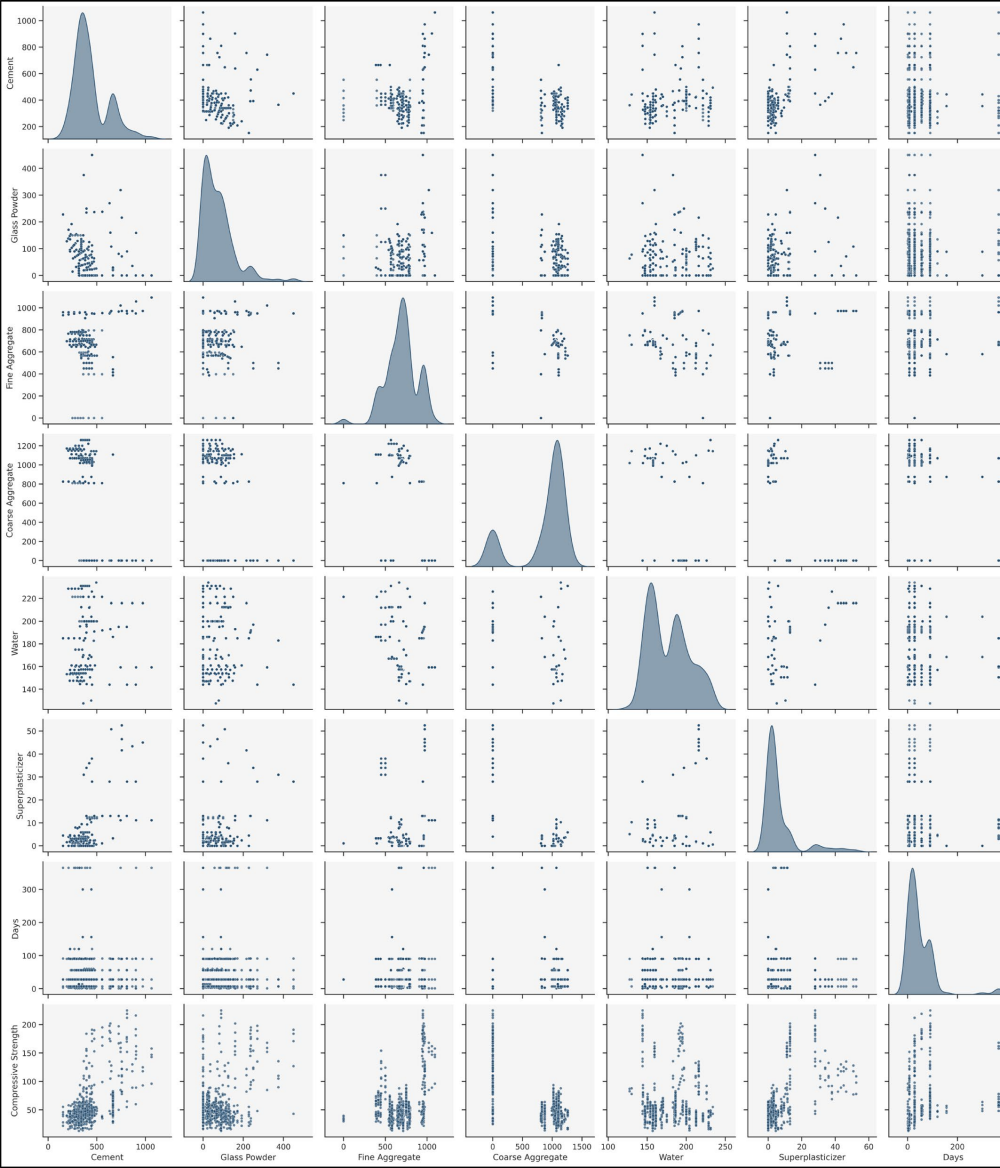
Pairwise Distribution of Input and Output Variables.

Moreover, the histogram plots (Fig. 2) depict the frequency distributions of each input variable and compressive strength. The cement and fine aggregate distributions show a near-normal spread, while glass powder and superplasticizer exhibit skewed distributions, suggesting varying replacement levels in different mix designs. The compressive strength histogram reveals a bimodal distribution, implying the presence of distinct strength categories in the dataset. The statistical analysis of input and output parameters is further validated by pair plot analysis (Fig. 3). It visually highlights potential correlations and distribution patterns, indicating possible nonlinear relationships between compressive strength and mixed components. Notably, cement and curing age exhibit a strong positive correlation with CS, while excessive water content shows a potential negative influence.

### Multicollinearity analysis

**Fig. 4:**
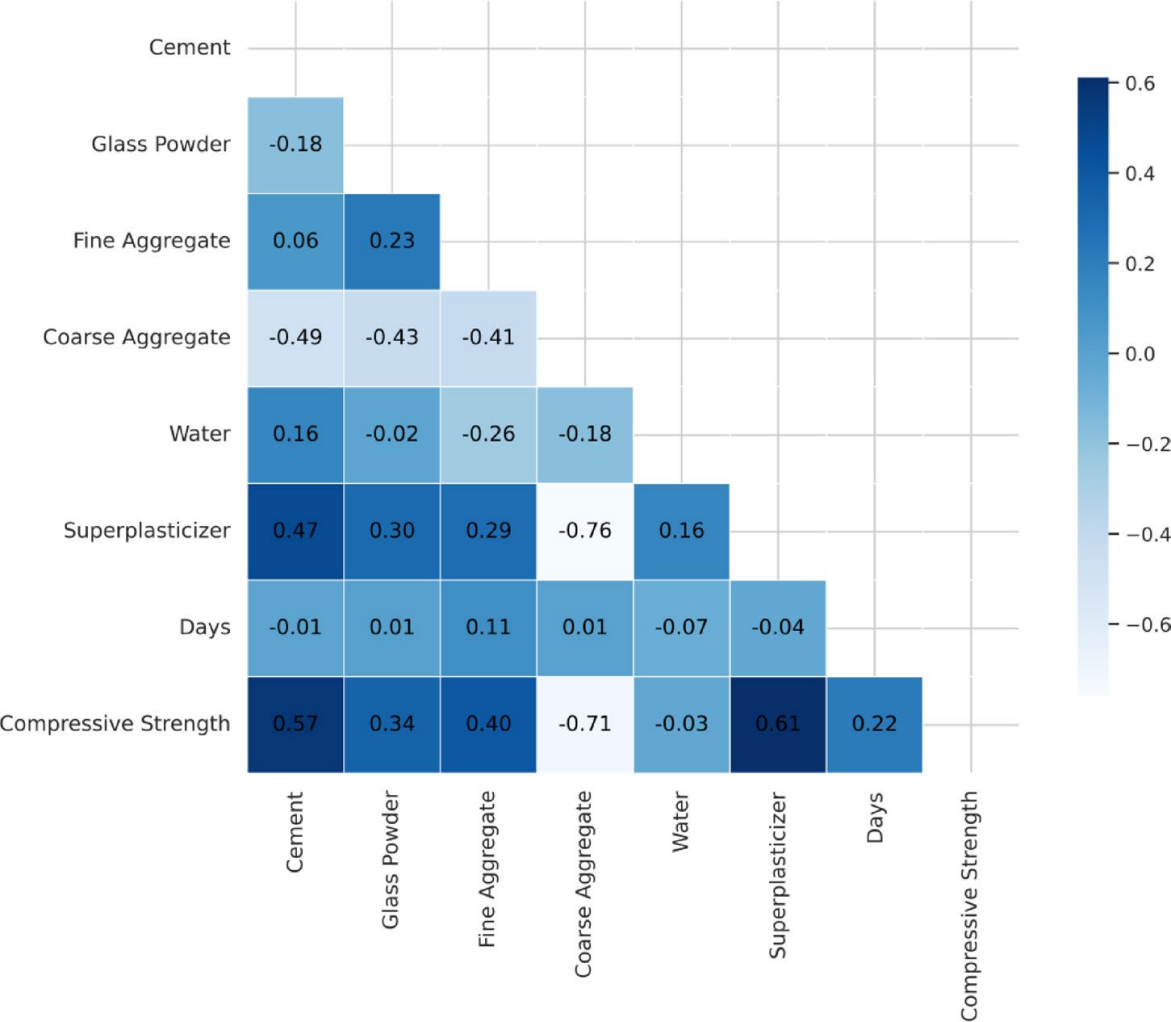
Pearson Correlation Heatmap - visualizes the correlation between input variables and compressive strength.

When independent variables in a regression model show a high degree of correlation, it is referred to as multicollinearity. This occurrence may skew the predicted coefficients and make the model less interpretable. In predictive modeling, multicollinearity poses a significant challenge as it can inflate variance, leading to unstable and unreliable predictions^67,68^. To assess multicollinearity in the dataset, Pearson correlation coefficients were computed between all independent variables, as illustrated in Fig. 4. The heatmap shows the correlation structure between the important mix design factors that affect the HSGPC’s CS. The analysis reveals that certain variables exhibit moderate to strong correlations with each other. Notably, cement content shows a positive correlation with compressive strength (0.57), indicating its fundamental role in improving concrete strength. Similarly, glass powder exhibits a moderate positive correlation (0.34) with CS, reinforcing its efficacy as a supplementary cementitious material. Conversely, coarse aggregate demonstrates a strong negative correlation (−0.71) with compressive strength, suggesting that higher coarse aggregate content may reduce strength due to weaker interfacial bonding and increased void content within the concrete matrix. SP content also displays a notable positive correlation (0.61) with CS.

Multicollinearity is considered critically high when the correlation coefficient exceeds 0.85, as it can disrupt machine learning models by introducing redundancy and instability in parameter estimation^69,70^. In this study, no extreme multicollinearity was detected among input features; however, moderate correlations between certain variables indicate the need for careful feature selection and engineering to improve models’ accuracy.

### Data preprocessing and splitting

The dataset was partitioned into training and testing sets in a 70:30 ratio, allocating 70% for model training and 30% for testing, to ensure effective model training and evaluation. To normalize the input variables, the Scikit-learn library’s Standard Scaler^71^ was used. To prevent models from being biased toward variables with bigger magnitudes, standardization converts the features to have zero mean and unit variance^72^.

### Outliers detection and analysis

Outliers are data points that deviate significantly from the overall distribution of the dataset and may arise from experimental variability, rare cases, or data recording inconsistencies^73^. In this study, outlier detection was performed using the Interquartile Range (IQR) method. According to this method, any value lying below $\:Q1-1.5\times\:IQR$ or above $\:Q3+1.5\times\:IQR$ is classified as an outlier, where Q1 and Q3 represent the 25 th and 75 th percentiles, respectively^74^.

**Fig. 5:**
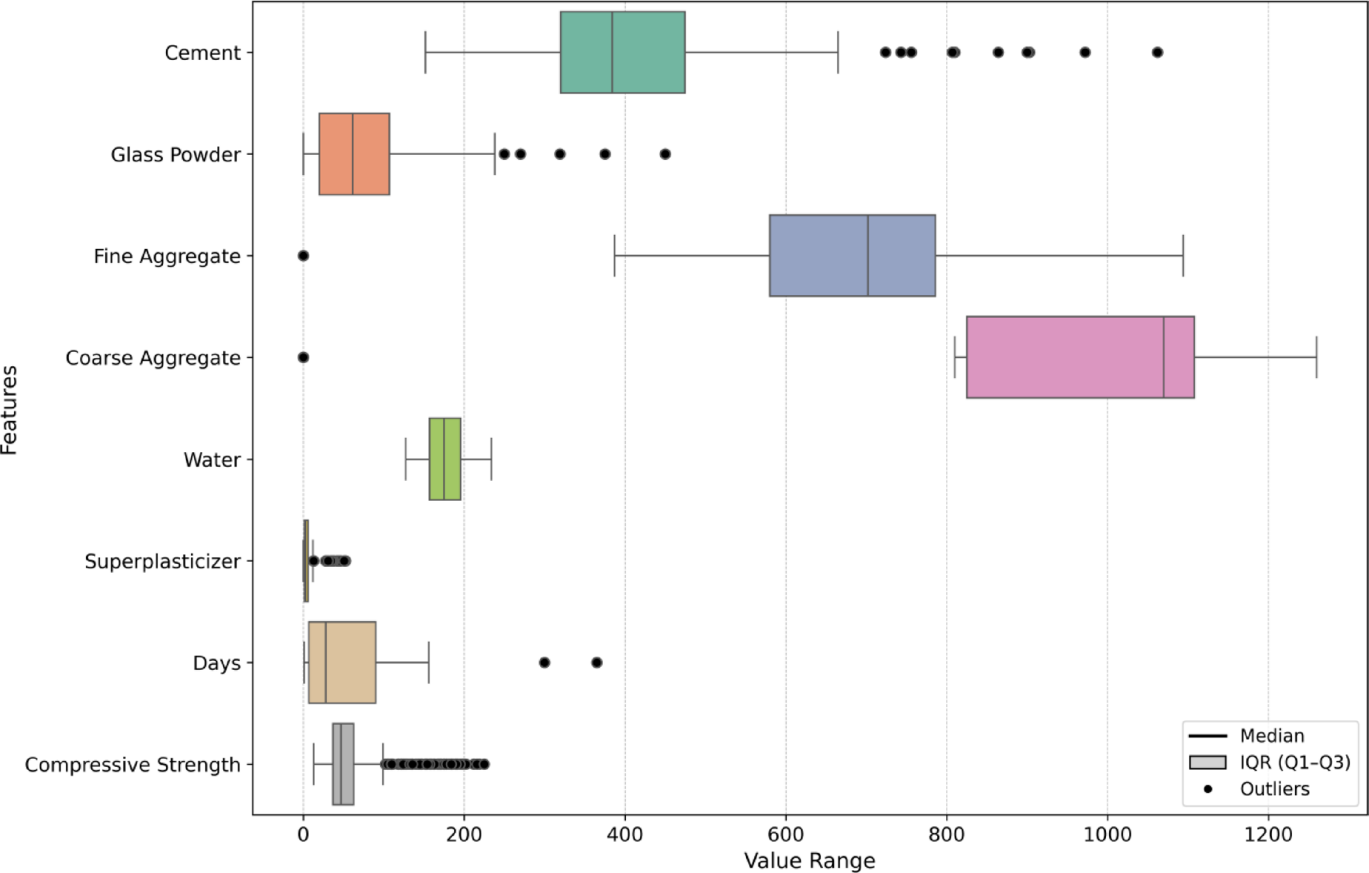
outlier distribution across input and output features using the IQR method.

To visualize and interpret the presence of outliers across all input variables and the target output (compressive strength), a horizontal boxplot was constructed, as shown in Fig. 5. The plot highlights the IQR spread and flags outliers as black circles beyond the whiskers. Among the eight features analyzed, coarse aggregate exhibited the highest proportion of outliers (18.56%), followed by superplasticizer (14.88%), and compressive strength (11.20%). Conversely, water content exhibited no outliers, indicating a more uniform distribution.

Rather than removing or replacing these values, all outliers were retained in the data set to preserve the natural variability and full range of mix design combinations present in HSGPC. Outliers in this context may represent deliberate, high-performance formulations or boundary conditions relevant to practical concrete applications.

### Models’ development

This section outlines the development and evaluation of predictive models for estimating the CS of HSGPC. Various individual machine learning models were implemented (KNN, RF, XGB) to assess their predictive accuracy and efficiency. To further enhance predictive performance, a hybrid approach was developed by integrating XGB with nature-inspired optimization algorithms, namely PSO, FA, and GWO whose details and their effectiveness are discussed in the following subsections.

#### K-Nearest neighbor

The KNN algorithm is a non-parametric, instance-based learning method that forecast the target variable based on the similarity of input features to its nearest neighbours in the dataset^75^. By analysing the nearest “k” data points and calculating their average response, KNN calculates the CS of additional data points. Distance measures like the Manhattan, Minkowski, and Euclidean distances are commonly used to quantify how similar two data points are^76^.

**Fig. 6:**
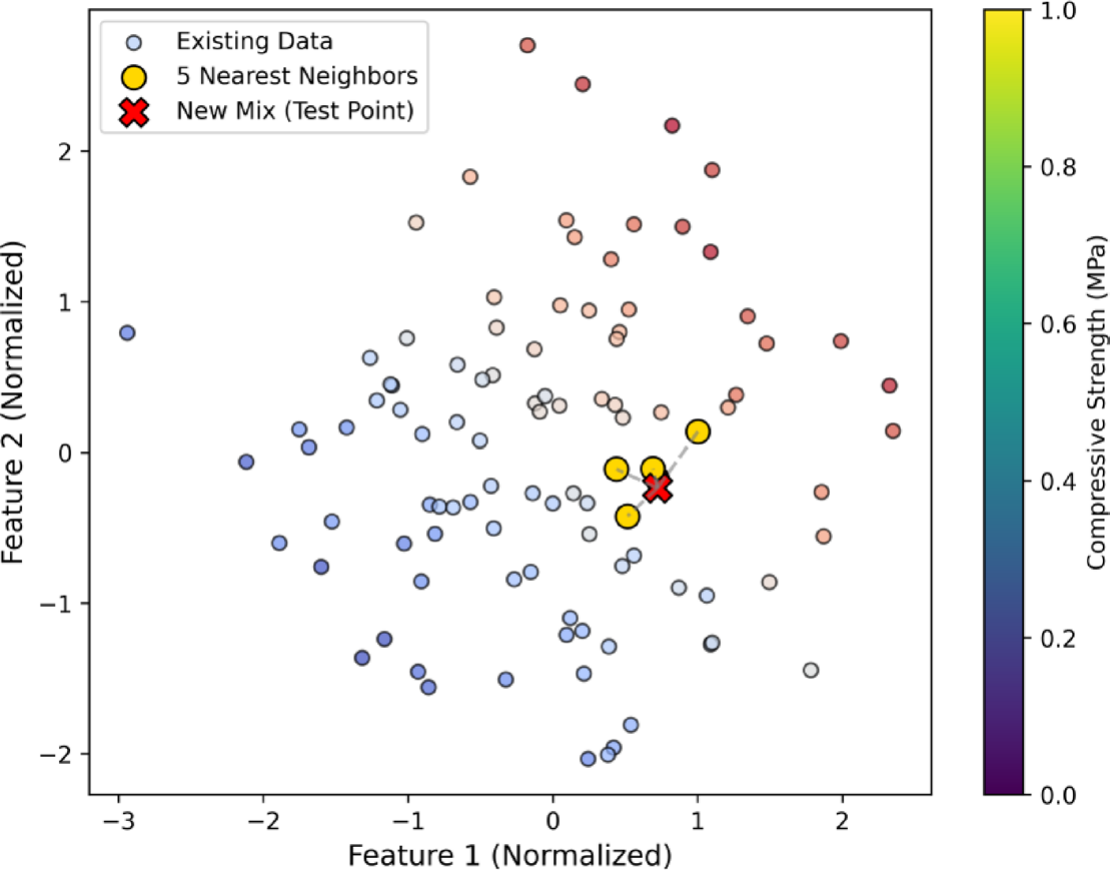
Visualization of K-Nearest Neighbors approach.

In this research, the Minkowski distance was selected as the similarity metric, with the power parameter *p* = 2, making it equivalent to the Euclidean distance. The optimal value of ‘k’ was determined based on empirical testing, and k = 5 was chosen as it provided the best trade-off between bias and variance, preventing overfitting while maintaining accurate predictions. The input features included cement content, glass powder percentage, fine and coarse aggregate proportions, water content, SP dosage, and curing age. Figure 6 illustrating the KNN approach, highlighting the five nearest neighbours (yellow) used to predict the CS of a new concrete mix (red).

#### Random forest

RF is an ensemble learning technique that builds multiple decision trees and aggregates their predictions to improve accuracy and reduce overfitting^77^. Given the variability of concrete mix parameters, especially coarse aggregates, RF is well-suited to predict compressive strength (CS). In this study, the RF Regressor employed 100 decision trees (n_estimators = 100), each trained on a random subset of data and input variables (bootstrap aggregation or bagging), to enhance generalization and minimize variance^78^. Final predictions were determined by averaging outputs from all trees, improving model stability and precision. A fixed random seed (random_state = 42) ensured reproducibility of results.

#### Extreme gradient boosting

Extreme Gradient Boosting (XGB) is an advanced gradient boosting algorithm known for its fast execution and accuracy^79^ developed by Chen and Guestrin^80^. It constructs sequential decision trees, each correcting the errors of the preceding one. Like RF, XGB effectively handles data with high variability by iteratively focusing on regions where previous models underperformed^81^. In this study, the XGB regressor was configured with 100 decision trees (n_estimators = 100) and a learning rate of 0.1, striking a balance between complexity and efficiency. XGB uses gradient boosting to sequentially learn from previous prediction errors and incorporates regularization techniques (L1 and L2) to prevent overfitting. Final predictions for CS were derived by aggregating weighted outputs from all trees.

#### Grey Wolf optimizer

**Fig. 7:**
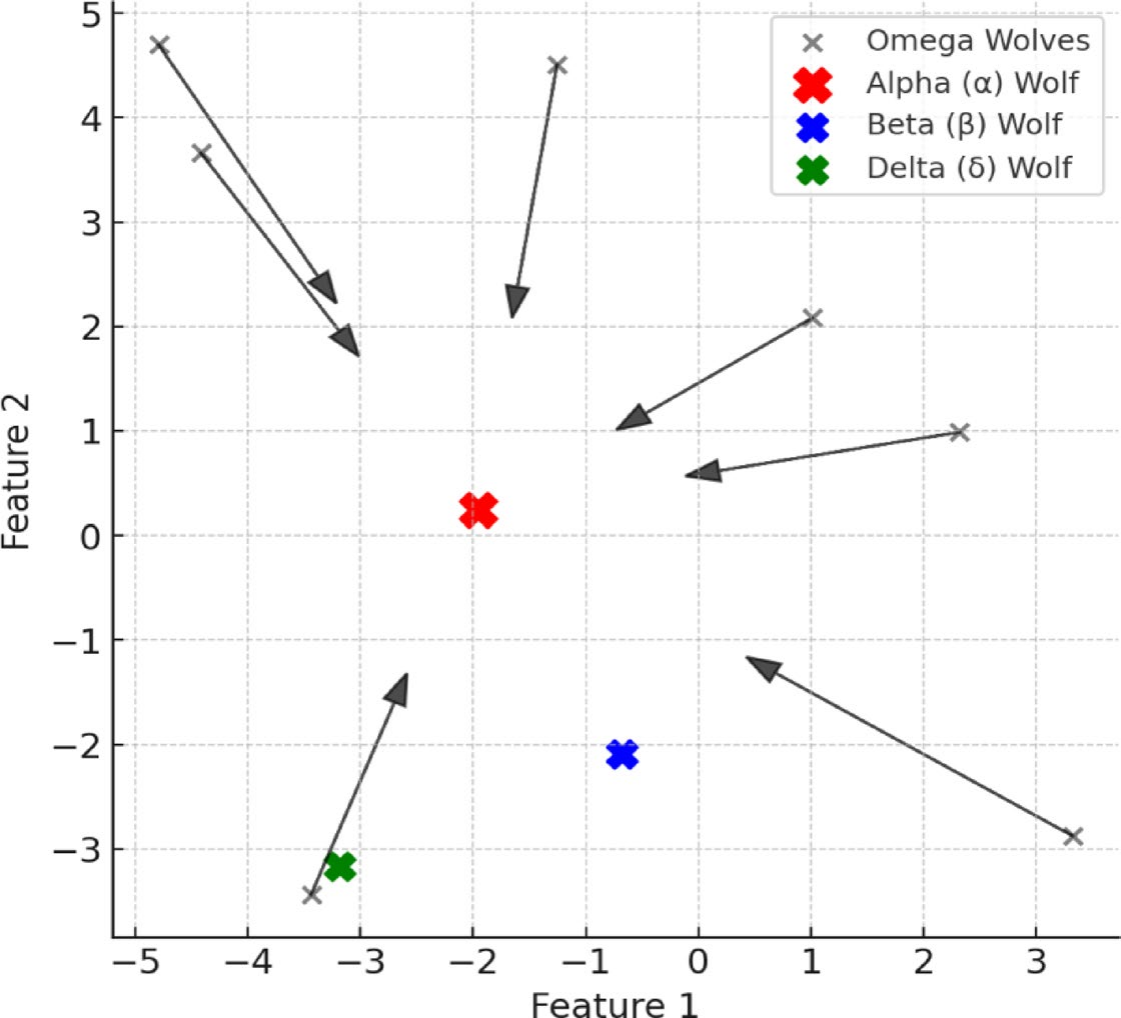
Visualization of GWO showing omega wolves moving toward the alpha (α), beta (β), and delta (δ) leaders in the optimization process.

The Grey Wolf Optimizer (GWO), proposed by Mirjalili in 2014^82^, is a nature-inspired metaheuristic known for its effectiveness, simplicity, and minimal parameter tuning^83^. Inspired by the hierarchical social structure and hunting strategies of grey wolves, GWO optimizes by iteratively updating three best solutions—alpha (α), beta (β), and delta (δ)—that guide the search process^84,85^. Other members (omega wolves, ω) adapt their positions based on these leading solutions, thus converging toward an optimal result. Due to its robustness, GWO has been applied widely in engineering, machine learning, bioinformatics, power flow control, scheduling, and cryptography^86–88^. Figure 7 visually represents the wolves’ movement patterns toward the optimal solution.

#### Particle swarm optimization

**Fig. 8:**
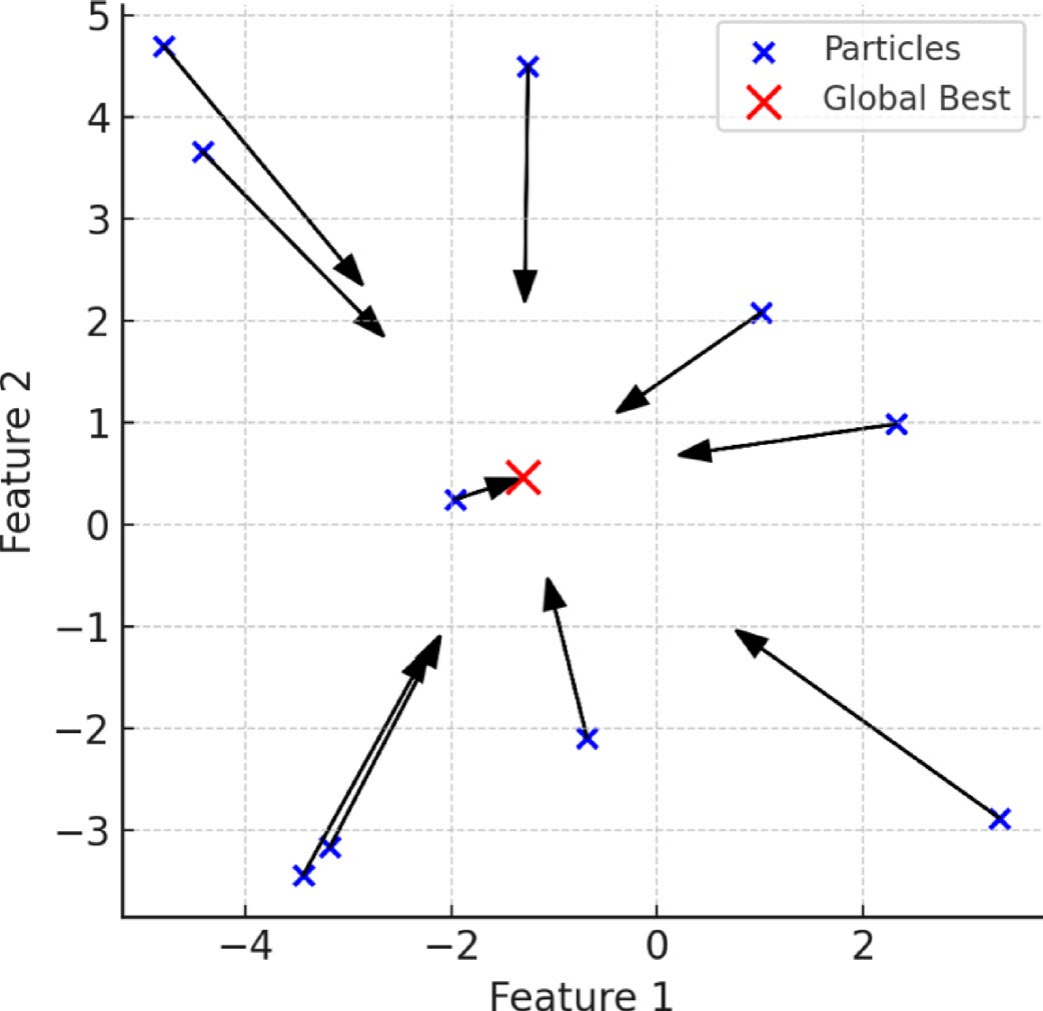
Visualization of PSO particles modifying their locations according to personal and global optimum solutions to achieve the best result.

PSO, inspired by swarm behaviours observed in nature, is a popular metaheuristic algorithm proposed by Eberhart and Kennedy in 1995^89,90^. It utilizes the collective intelligence of particles (candidate solutions) to effectively explore and exploit the search space. Each particle updates its position based on its personal best solution (pBest) and the global best solution (gBest) identified by the swarm, guided by inertia weight, cognitive, and social parameters^91,92^. PSO is well-suited to dynamic optimization problems due to its minimal constraints on objective function continuity, straightforward implementation, and computational efficiency^93^. Figure 8 illustrates how particles iteratively move toward the optimal solution through swarm intelligence.

#### Firefly algorithm

**Fig. 9:**
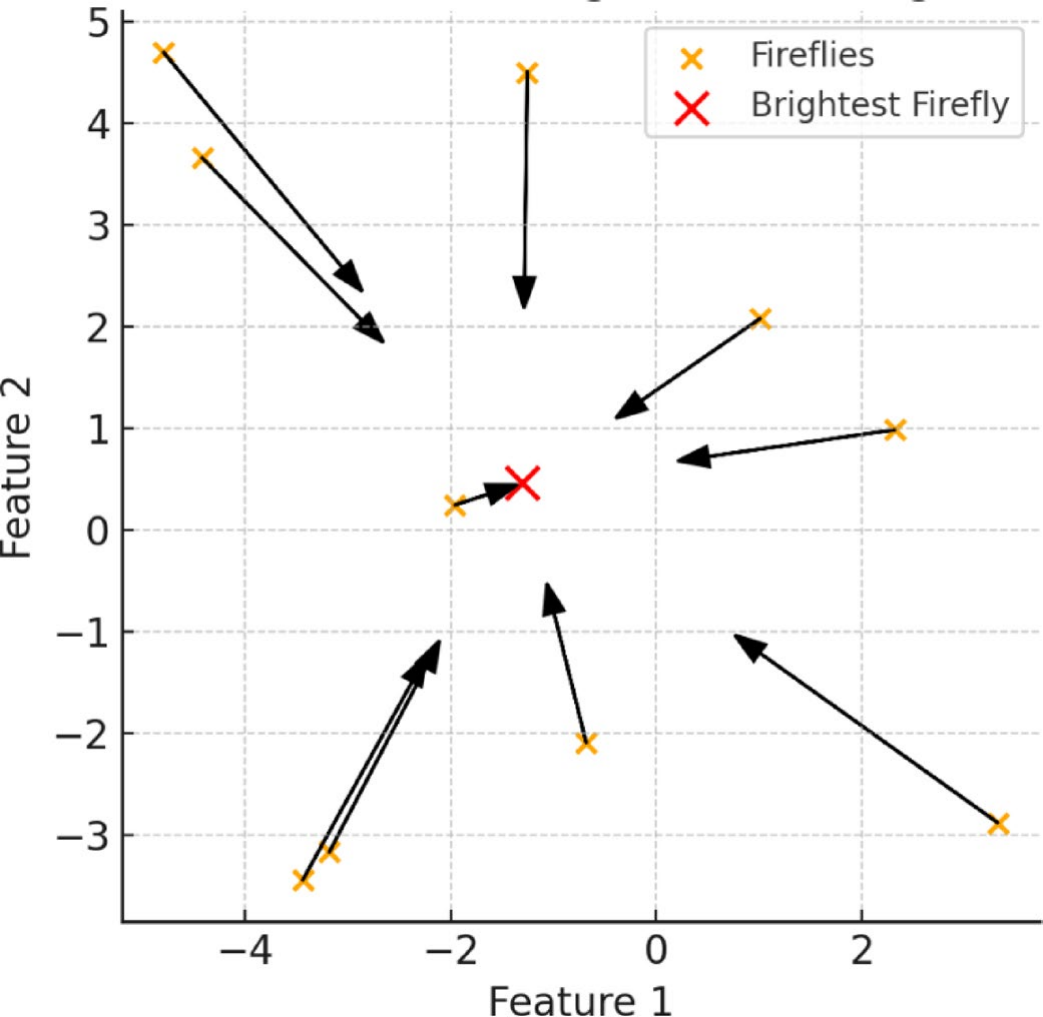
Visualization of FA fireflies moving toward the brightest firefly, mimicking swarm intelligence for optimization.

FA is a nature-inspired optimization technique simulating the bioluminescent attraction behaviours of fireflies, effectively balancing global and local searches^94,95^. In FA, less bright fireflies move toward brighter ones based on their brightness (fitness), intelligently guiding the search process toward optimal solutions^96^. The attraction intensity diminishes with distance, ensuring diverse exploration while preventing stagnation at local optima through random movements if no brighter neighbours are found. Due to its capability in addressing high-dimensional and nonlinear optimization problems, FA has been successfully applied in engineering design, machine learning, and multi-criteria decision-making^96^. Figure 9 visually illustrates the FA mechanism, depicting fireflies moving toward the brightest solutions.

### Evaluation metrics

- **R² (Coefficient of Determination)**: Determines the proportion of the dependent variable’s variance that the model can explain, ranging from -∞ to 1, where 1 indicates a perfect fit.

$$\:{R}^{2}=\:1-\frac{{{\Sigma\:}}_{i=1}^{n}{\left({x}_{i}-{\widehat{x}}_{i}\right)}^{2}}{{{\Sigma\:}}_{i=1}^{n}{\left({x}_{i}-\stackrel{-}{x}\right)}^{2}}$$

- **RMSE (Root Mean Squared Error)**: Computes the root mean square deviation between the input and output data, represented as the square root of the average squared difference, with units matching the target variable.

$$\:RMSE=\sqrt{\frac{1}{n}{\sum\:}_{i=1}^{n}{\left({x}_{i}-{\widehat{x}}_{i}\right)}^{2}}$$

- **NRMSE (Normalized RMSE)**: Normalizes RMSE by the range or mean of the target variable, providing a unitless measure of error.

$$\:NRMSE=\:\frac{RMSE}{{x}_{max}-{x}_{min}}$$

- **MSE (Mean Squared Error)**: Computes the mean squared deviation between the input and output data.

$$\:MSE=\:\frac{1}{n}{\sum\:}_{i=1}^{n}{\left({x}_{i}-{\widehat{x}}_{i}\right)}^{2}$$

- **MAE (Mean Absolute Error)**: Assesses the mean absolute discrepancies between expected and actual values, with units matching the target variable.

$$\:MAE=\:\frac{1}{n}{\sum\:}_{i=1}^{n}\left|{x}_{i}-{\widehat{x}}_{i}\right|$$

- **MAPE (Mean Absolute Percentage Error)**: Denotes the mean percentage discrepancy between forecasted and actual values, providing a unitless measure.

$$\:MAPE=\:\frac{100\%}{n}{\sum\:}_{i=1}^{n}\left|\frac{{x}_{i}-{\widehat{x}}_{i}}{{x}_{i}}\right|$$

## Results and discussion

### Performance evaluation

**Fig. 10:**
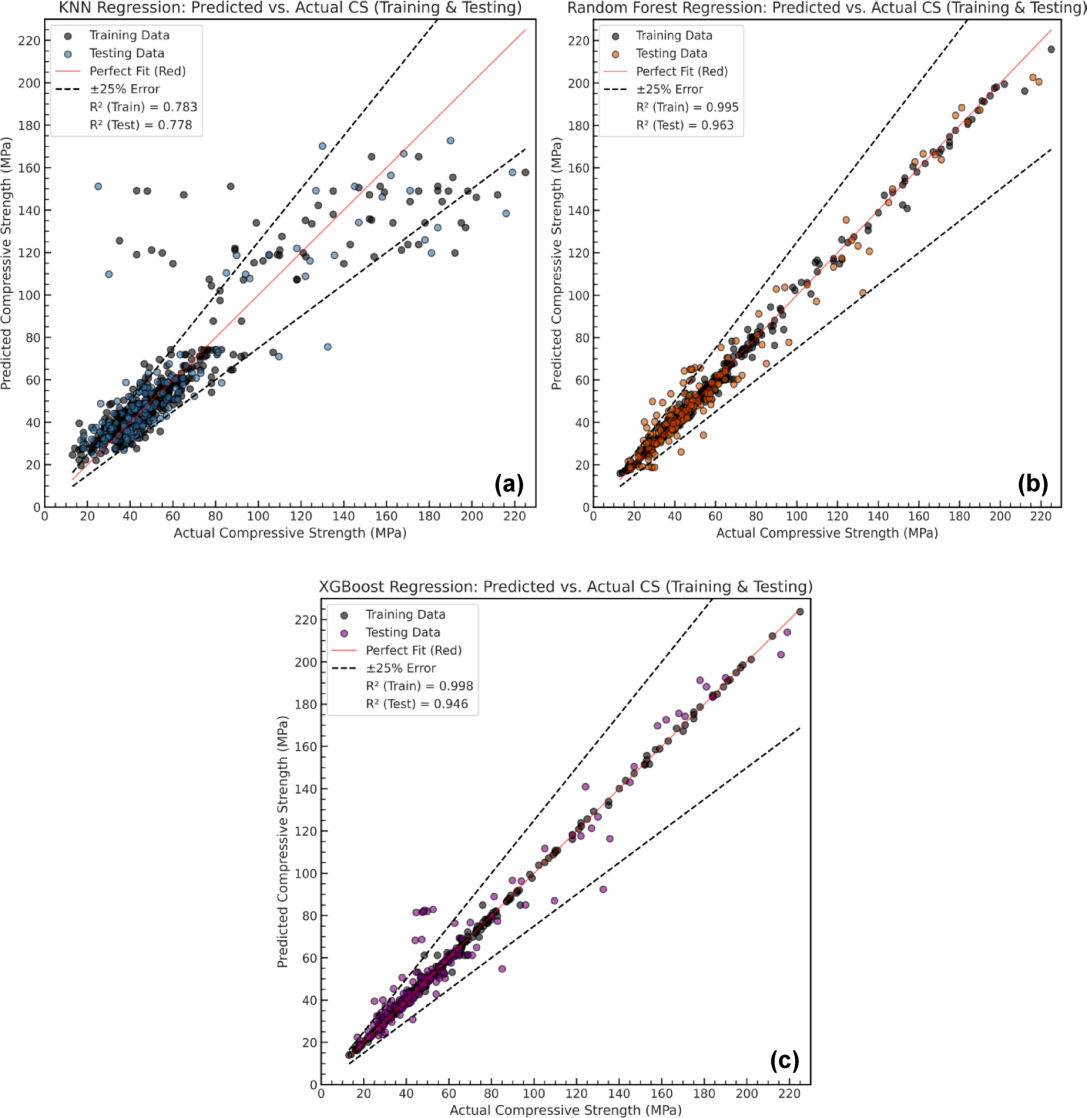
Predicted vs. Actual compressive strength for different standalone models: (**a**) KNN, (**b**) RF, and (**c**) XGB.

For both training and testing phase, R^2^ values were used to evaluate the forecasting efficacy of the generated models, which included KNN, RF, and XGB. The findings, which are shown in Fig. 10 (a–c), demonstrate how well each model can learn and generalize the link between compressive strength and mix design parameters. The KNN model exhibited an R² value of 0.783 on the training dataset, indicating that it was able to capture a substantial portion of the data variability. However, its performance on the test dataset dropped to 0.778, signifying a moderate reduction in generalization capability.

Moreover, both RF and XGB demonstrated excellent predictive capabilities, achieving high R² values on both training and testing datasets. RF attained an R² of 0.995 (train) and 0.963 (test), while XGB recorded 0.999 (train) and 0.946 (test). Their high-test performance highlights their ability to generalize well, benefiting from ensemble learning and boosting techniques that reduce variance and improve model stability. As seen in Fig. 10 (b, c), both models align closely with actual CS values, with minimal deviations beyond the ± 25% error margin.

The models’ detailed evaluation metrics, such as MSE, RMSE, MAE, and MAPE for both training and testing datasets, are shown in Table 4. The RF model demonstrated the best predictive accuracy, with the lowest MSE (8.118 train, 56.851 test), RMSE (2.849 train, 7.540 test), and MAE (1.903 train, 5.330 test). XGB came in second regarding MSE, with slightly higher error values but still strong generalization in terms of other metrics. The KNN model, however, showed the highest error rates, particularly in testing (MSE: 343.589, RMSE: 18.536, MAE: 10.512), reflecting its lower reliability compared to the tree-based models. The MAPE values confirm this trend, with XGB achieving the lowest error percentage, followed by RF, while KNN demonstrated the highest deviation. These results reinforce the effectiveness of ensemble-based approaches (RF and XGB) over distance-based regression techniques (KNN) to forecast the CS of HSGPC.

**Table 4:** Evaluation metrics (Test, Train) of KNN, RF and XGB.

Evaluation Metrics	KNN	RF	XGB
R² (Train)	0.783	0.995	0.998
R² (Test)	0.778	0.963	0.946
MSE (Train)	324.250	8.118	2.824
MSE (Test)	343.589	56.851	83.950
RMSE (Train)	18.007	2.849	1.680
RMSE (Test)	18.536	7.540	9.162
MAE (Train)	9.992	1.903	1.004
MAE (Test)	10.512	5.330	5.154
MAPE (%) (Train)	18.054	3.735	2.109
MAPE (%) (Test)	21.910	11.378	10.062

### Hybrid model optimization and performance analysis

To further optimize and enhance the test performance of XGB, the model was integrated with nature-inspired optimization algorithms, namely PSO, FA, and GWO. These metaheuristic approaches were used to adjust XGB’s hyperparameters to increase its capacity for generalization and prediction accuracy.

The optimal hyperparameters, including the number of estimators, max depth, learning rate, subsample ratio, and column sample by tree ratio, were consistently identified for each optimization method. Table 5 presents a summary of the hyperparameters optimized by PSO, FA, and GWO.

**Table 5:** Optimized hyperparameters for XGB using PSO, FA, and GWO.

Hyperparameters	PSO Optimized Values	FA Optimized Values	GWO Optimized Values
Number of estimators	348	373	500
Maximum Depth	10	13	4
Learning rate	0.1422	0.1308	0.1621
Sub sample size	0.8254	0.7657	0.8876
Column sample by tree ratio	0.5259	0.5372	0.5197

**Fig. 11:**
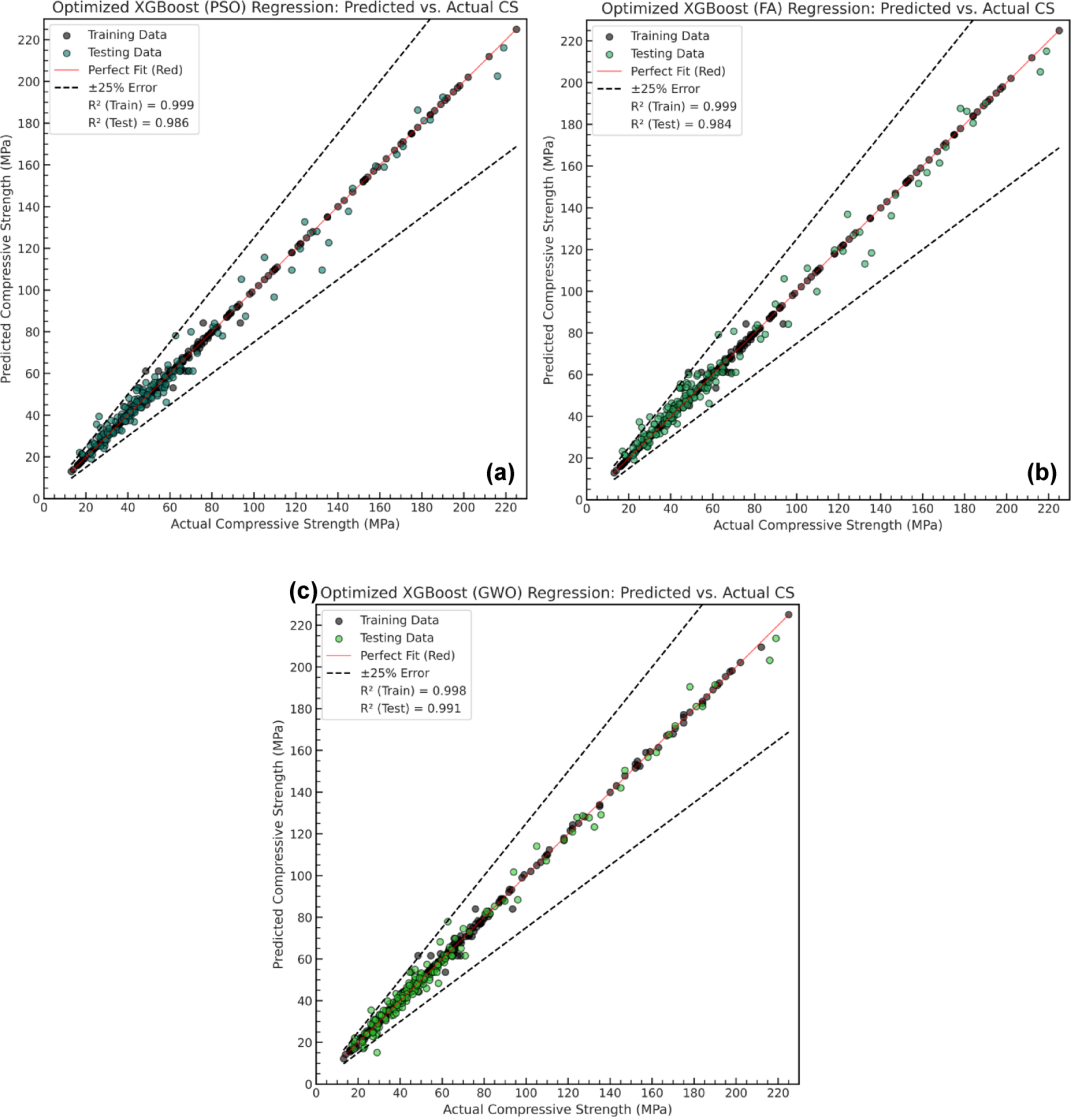
Predicted vs. Actual compressive strength for hybrid models: (**a**) XGB-PSO, (**b**) XGB-FA, and (**c**) XGB-GWO.

The optimized XGB models were evaluated using R² scores and multiple error metrics to assess their effectiveness in predicting compressive strength. The optimized models achieved R² values of 0.986 (XGB-PSO), 0.984 (XGB-FA), and 0.991 (XGB-GWO) on the test dataset, demonstrating enhanced predictive capability. Figure 11 (a-c) illustrate the regression plots for each optimized model, comparing predicted and actual compressive strength values. The optimized models exhibit tighter clustering along the perfect fit line, with fewer data points deviating beyond the ± 25% error margins.

**Fig. 12:**
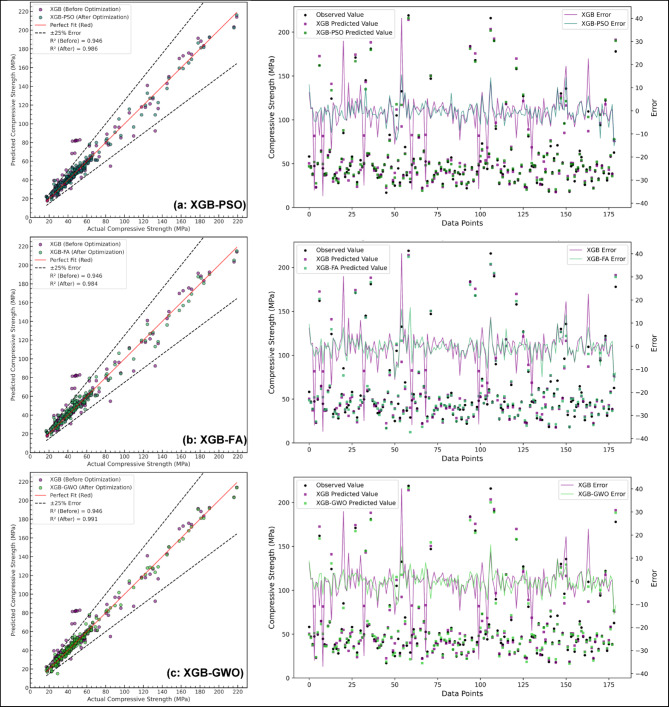
Test phase comparison of XGB performance before and after optimization using (**a**) PSO, (**b**) FA, and (**c**) GWO, based on regression R² values and error analysis.

Additionally, Fig. 12 (a-c) presents a comprehensive comparison of the XGB model before and after optimization, highlighting the performance of each hybrid model relative to the baseline XGB model based on testing phase. Both regression plots and error analysis visualizations provide insights into the distribution of errors across data points. The results indicate that the optimized models significantly enhanced predictive performance, improved reliability and reduced prediction deviations in the modified models.

**Fig. 13:**
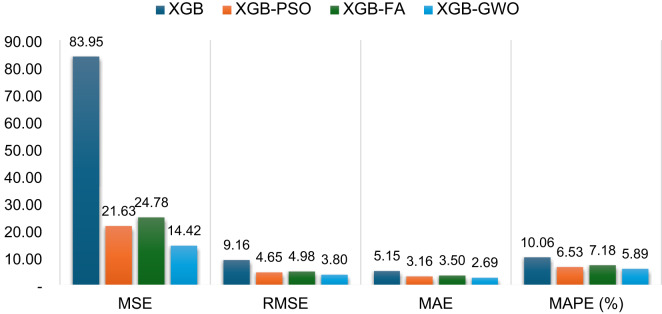
Comparative performance evaluation of XGB before and after optimization (Test Phase).

In addition to the regression and error plots, Fig. 13 provides a comparative bar chart illustrating the reduction in error metrics (MSE, RMSE, MAE, and MAPE) after optimization. The optimized models demonstrated a substantial decline in MSE, with XGB-GWO achieving the lowest value (14.42), followed by XGB-PSO (21.63) and XGB-FA (24.78). Similarly, RMSE, MAE, and MAPE were significantly reduced across all optimized models, reaffirming the effectiveness of the applied metaheuristic techniques in enhancing the model’s predictive performance. Among the three optimization techniques, XGB-GWO demonstrated superior test performance, attaining the greatest R² score (0.991) and the least MSE (14.42). These findings are in line with previous studies^97,98^ as GWO balances exploration and exploitation effectively. For instance, A study by Ӧzyüksel Çiftçioğlu et al.^99^ developed a GWO-XGB model for UHPC incorporating nano- and micro-materials, achieving R² values of 0.984 and 0.948, respectively, for CS prediction. Similarly, Parhi et al.^73^ implemented a hybrid ensemble model using XGB/GWO-optimized RF, NN, and MARS to predict the strength of geopolymer concrete, reporting an R² of 0.938 on the test set. In another work, Zhang et al.^98^ proposed a GWO-optimized RF–XGB stacking model for landscape geopolymer concrete, achieving R² values of 0.983 and 0.981 with significantly reduced RMSE. These findings affirm that the integration of metaheuristic optimization, particularly GWO, enhances the reliability of ML models. Compared to these studies, the current work’s XGB-GWO model for HSGPC concrete yields comparable or better accuracy. Moreover, the ML models demonstrated strong generalization capability and predictive accuracy despite the presence of outliers (Fig. 5), as evidenced by the performance metrics. This supports the robustness of the models and justifies the retention of the full dataset for training and evaluation.

### Taylor diagram

**Fig. 14:**
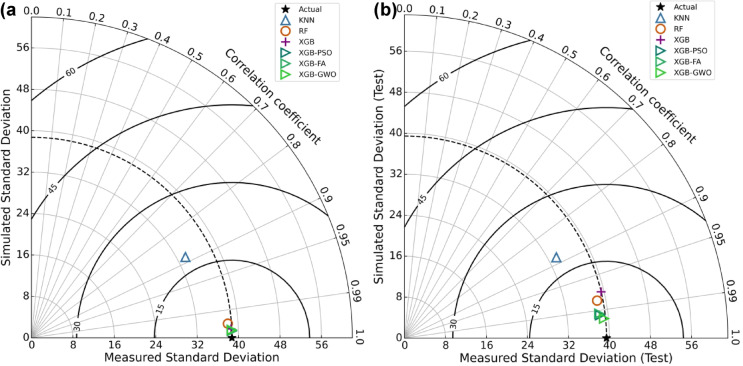
Taylor diagrams for (**a**) training and (**b**) testing phases showing the comparative performance of all models.

Figure 12(a, b) illustrates Taylor diagrams that compare the performance of machine learning models (KNN, RF, XGB) and hybrid-optimized XGB models (XGB-PSO, XGB-FA, XGB-GWO) in predicting the compressive strength of HSGPC. These diagrams simultaneously present the correlation coefficient, standard deviation, and root mean square error (RMSE), where the contour lines indicate the centered RMSE between predicted and actual values. In the training phase (Fig. 14(a)), XGB and its optimized variants exhibit near-perfect agreement with the observed standard deviation and the highest correlation coefficients (> 0.999), indicating excellent model fitting. In the testing phase (Fig. 14(b)), the XGB-GWO model achieved a highest correlation coefficient of 0.9952, with a predicted standard deviation of 39.143 MPa, closely matching the observed standard deviation of 39.446 MPa. This proximity to the reference point is also associated with the lowest RMSE contour level, aligning with the model’s recorded test RMSE of 3.80 MPa. In contrast, the KNN model showed a notably lower correlation (*R* = 0.882) and a standard deviation of 33.570 MPa, indicating weaker agreement and higher prediction error. These Taylor diagrams further validate and visually compare the findings of all models, reinforcing the enhanced prediction capability of hybrid-optimized XGB models especially the GWO-enhanced variant.

### SHAP analysis

The SHapley Additive Explanations (SHAP), a complex method for elucidating the outputs of any machine learning model, facilitates the comprehension of complex machine learning models^100^. By allocating relevance values to each feature according to how it contributes to a particular prediction, SHAP enables researchers to comprehend how the trained model makes decisions. Based on game theory, the method calculates the contribution of each characteristic in the dataset to the overall prediction, in a manner like how a player’s contribution is assessed in a cooperative game^78,101^.

To examine the impact of cement, glass powder, fine and coarse aggregates, water, superplasticizer, and curing age on compressive strength predictions, SHAP was used to the improved XGB model in this study. Feature significance was shown using the SHAP summary plot, which displays the total effect of every variable. To evaluate the interplay between distinct characteristics and their impact on CS, the SHAP dependency plot was also produced.

#### SHAP summary and feature importance

**Fig. 15:**
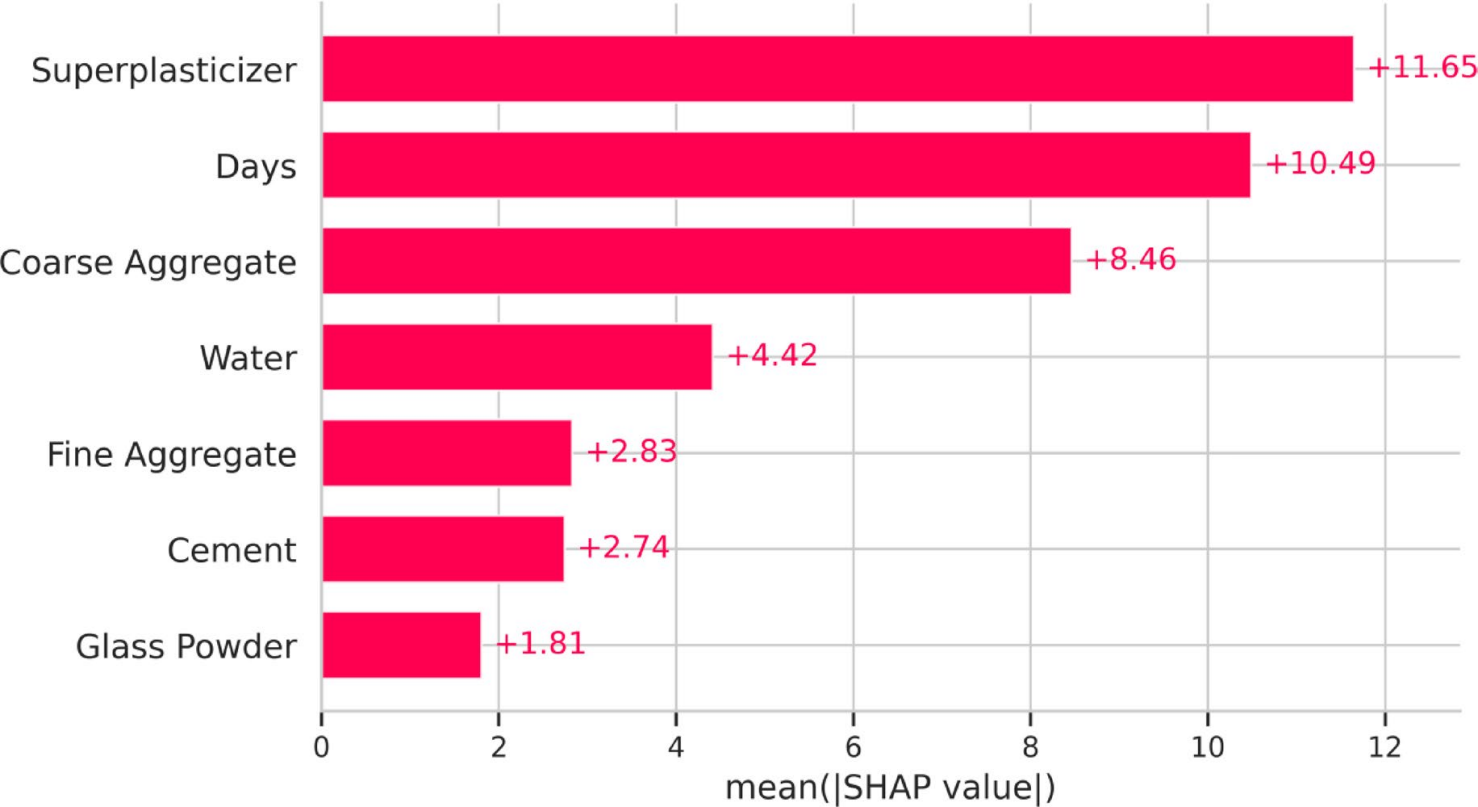
SHAP feature importance plot showing the average absolute SHAP values.

The influence of input parameters on CS prediction is ranked using the SHAP feature importance ranking (Fig. 15), curing days (+ 10.49), coarse aggregate (+ 8.46), water content (+ 4.42), fine aggregate (+ 2.83), cement (+ 2.74), and glass powder (+ 1.81) are the next most influential factors, followed by SP dosage with a SHAP value of + 11.65.

**Fig. 16:**
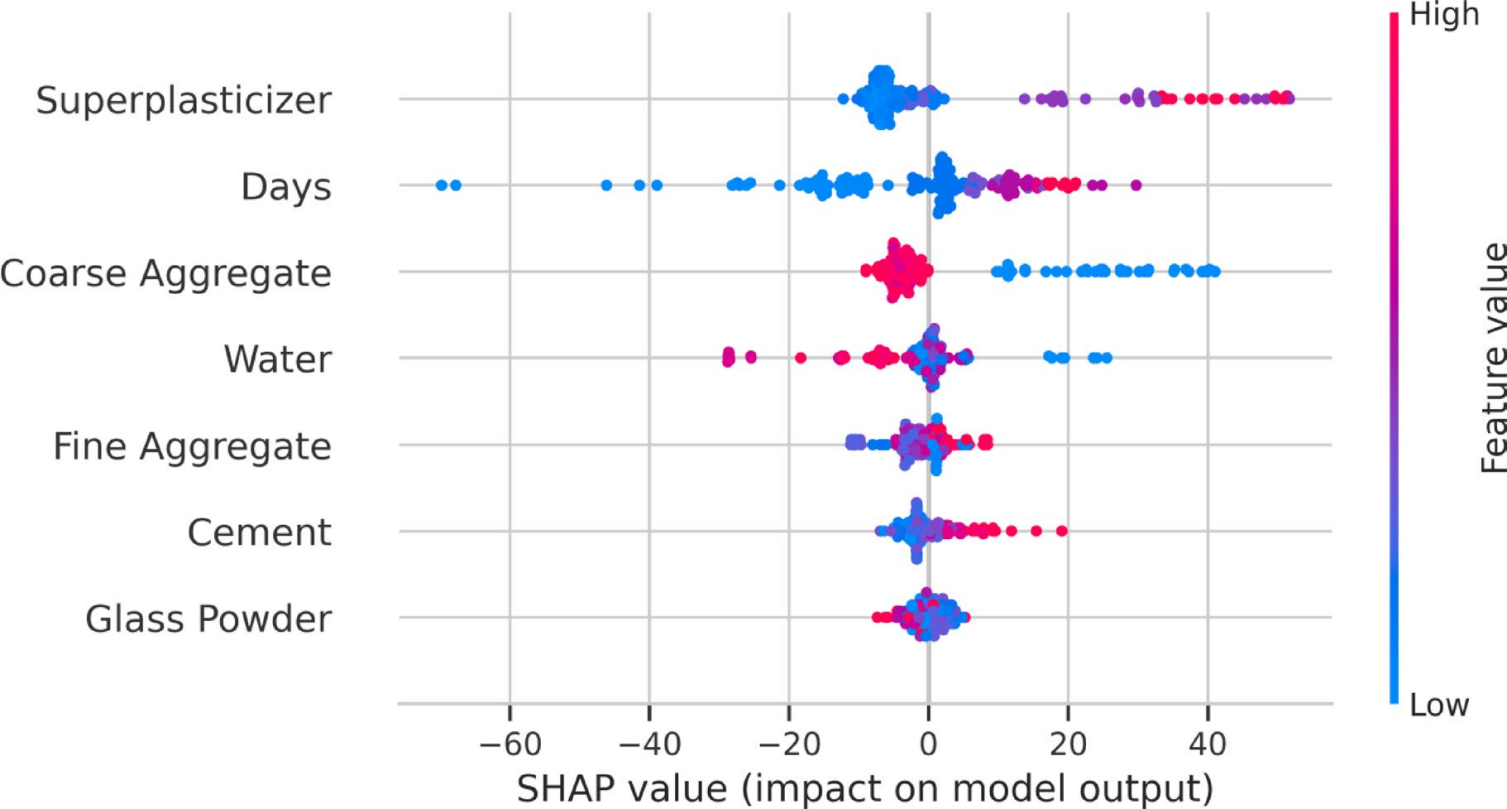
SHAP summary plot illustrating the distribution of SHAP values for each input variable.

The SHAP summary plot (Fig. 16) further substantiates these findings. Interestingly, coarse aggregate showed a high influence on the negative side, suggesting that excessive amounts may reduce strength, possibly due to insufficient paste-to-aggregate bonding or an increase in interfacial transition zone (ITZ) defects. Conversely, curing days significantly enhance compressive strength, reinforcing the well-established role of extended hydration in improving concrete performance. The notable impact of superplasticizer dosage indicates its role in optimizing workability and enhancing particle dispersion, leading to improved strength outcomes in HSC.

Additionally, glass powder exhibited the lowest SHAP impact, which can be attributed to its sequential replacement with cement rather than direct enhancement of mechanical strength. However, its contribution remains positive, aligning with its pozzolanic activity and potential to improve long-term strength development. The moderate influence of cement and fine aggregate highlights their essential, yet balanced, contribution to the overall mix design.

#### SHAP dependence analysis for individual features

To further investigate the influence of individual material components on compressive strength, SHAP dependence plots were generated, providing deeper insights into how variations in mix proportions affect strength development in high-strength glass powder concrete.

**Fig. 17:**
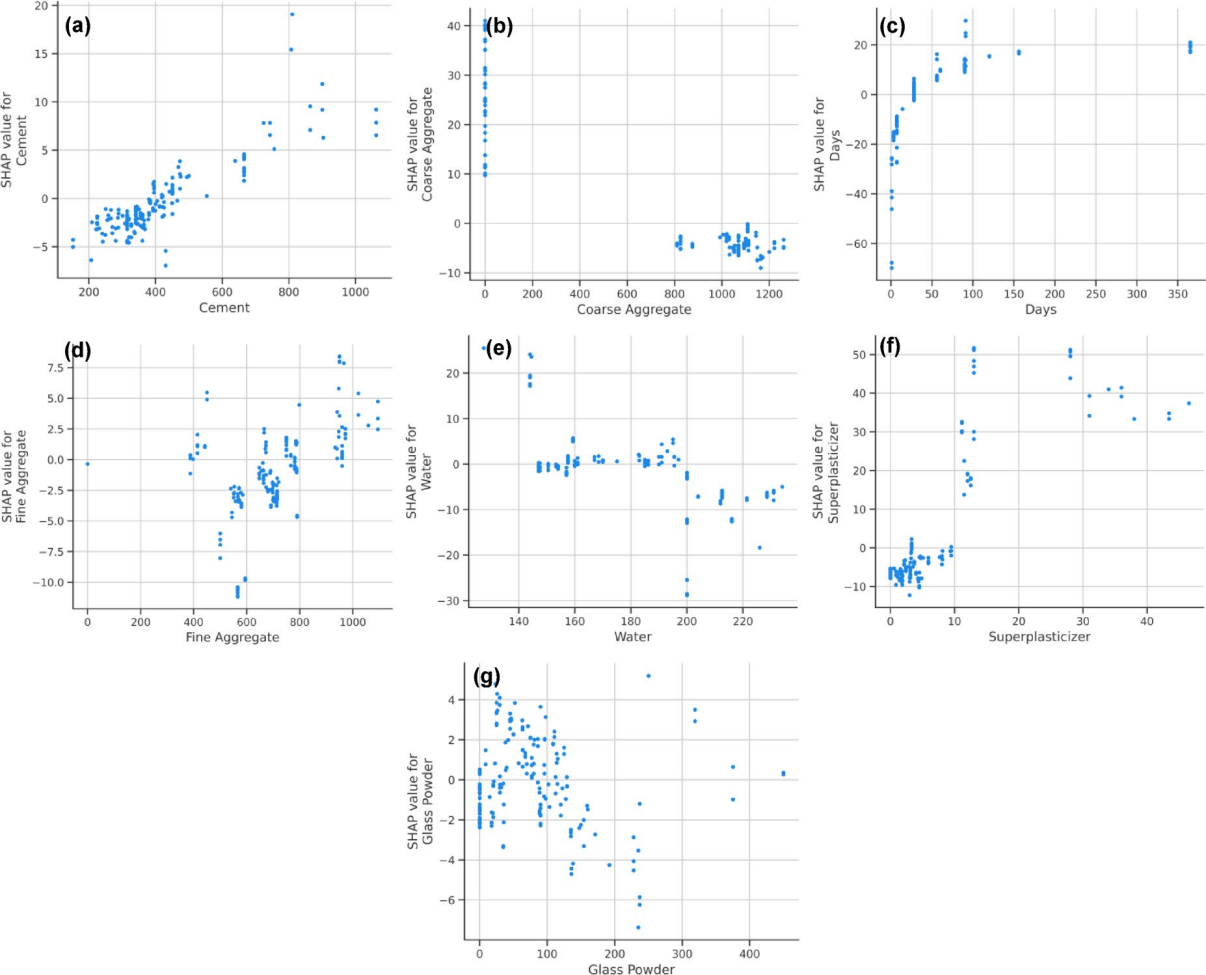
SHAP dependence plots illustrating the impact of individual features on compressive strength predictions: (**a**) Cement, (**b**) Coarse Aggregate, (**c**) Curing Days, (**d**) Fine Aggregate, (**e**) Water, (**f**) Superplasticizer, and (**g**) Glass Powder.

The first important parameter, cement has a robust positive association with CS, underscoring its function as the principal binder in concrete. As observed in the SHAP dependence plot (Fig. 17a), an increase in cement content generally leads to a higher CS. However, beyond a certain threshold, the effect diminishes, likely due to inefficient hydration at excessive cement levels, which can contribute to shrinkage cracks and increased brittleness. This trend highlights the necessity of maintaining an optimal cement proportion to achieve maximum strength without compromising durability.

There is a non-linear tendency in the association between CS and coarse aggregate content (Fig. 17b). Strength is increased by increasing coarse aggregate at moderate levels because it improves load distribution within the matrix. However, an excessive amount of coarse aggregate results in a little decrease in compressive strength, which may be caused by decreased matrix cohesion and impaired paste-aggregate bonding. Moreover, curing days, as shown in Fig. 17c, demonstrate a strong positive influence on compressive strength. The SHAP analysis confirms that prolonged curing significantly enhances strength development by allowing extended hydration reactions to take place. This aligns with well-established principles of concrete technology, where longer curing periods improve microstructural densification, reduce porosity, and enhance durability.

Fine aggregate and water content also exhibit moderate influence on compressive strength Fig. 17d and e). While fine aggregate contributes to the overall packing density and strength of the mix, its effect is less pronounced compared to coarse aggregate and cement. Conversely, water content is essential for workability; nevertheless, an excess of water results in enhanced porosity and diminished strength. The SHAP dependency plot indicates that sustaining an ideal water-to-cement ratio is crucial for achieving a balance between workability and strength in high-strength concrete. Superplasticizer dosage follows a distinctive trend, as seen in Fig. 17f, where an optimal dosage enhances workability and contributes to higher compressive strength. However, excessive superplasticizer can disrupt particle cohesion, leading to segregation and reduced strength development.

Glass powder, despite having the lowest SHAP influence, still plays a positive role in strength development (Fig. 17g). Unlike other mix components, its lower influence can be attributed to its sequential replacement with cement, meaning its effect is moderated by the presence of the primary binder. The SHAP dependence plot suggests that glass powder contributes to long-term strength enhancement through pozzolanic activity, improving the overall sustainability of the concrete mix without significantly altering its early-age performance.

### Partial dependence plot (PDP) analysis

The Partial Dependence Plots (PDPs) further validate the feature importance and trends observed in the SHAP analysis. These are low-dimensional graphical representations of prediction functions that show how the result and relevant predictors relate to one another. Partial dependency plots validate the use of explanatory variables for prediction and enable model review^102^.

**Fig. 18:**
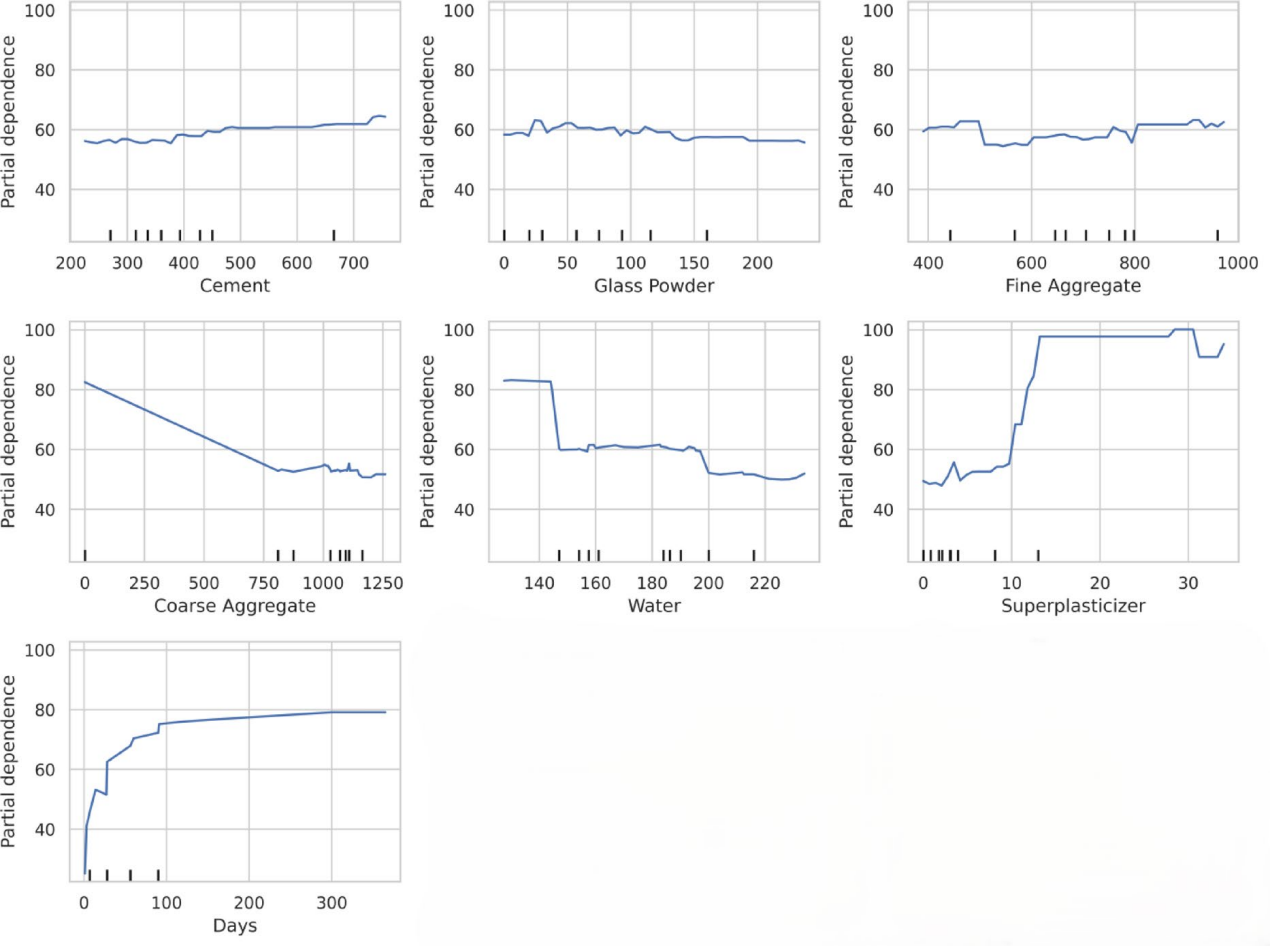
Partial Dependence Plots (PDP) showing the influence of individual features on compressive strength predictions.

The results (Fig. 18) confirm that cement, curing days, fine aggregate, and superplasticizer positively contribute to compressive strength, reinforcing their role in enhancing concrete performance. Cement and curing days show a strong correlation with strength gain, while fine aggregate and superplasticizer exhibit moderate yet beneficial effects. The effect of glass powder remains stable, further supporting its supplementary role as a cement replacement without significantly impacting strength.

Conversely, coarse aggregate and water content display a negative influence at higher proportions, aligning with the SHAP findings. Excessive coarse aggregate disrupts the paste-aggregate bond, while increased water content reduces matrix density, leading to lower compressive strength. There are well established concrete engineering theories that excessive coarse aggregate content reduces concrete compressive strength primarily by altering the paste-to-aggregate ratio and weakening the Interfacial Transition Zone (ITZ). An overly high aggregate volume results in insufficient cementitious paste, leaving uncoated aggregate surfaces and internal voids that diminish concrete density and strength^103^. Additionally, excessive coarse aggregate enlarges and overlaps the ITZ regions, inherently porous zones with higher local water-to-cement ratios. These enlarged ITZs promote stress concentration and initiate cracking under lower stress, further compromising strength^104,105^. In this regard, Malkawi et al.^104^ found that a moderate coarse aggregate ratio (about 55–65% of total aggregate by volume) yielded the highest compressive strength in a high-strength fly ash geopolymer concrete, whereas mixes with higher coarse aggregate content showed significantly lower strength along with increased porosity. Furthermore, an excessively coarse aggregate fraction impairs workability and compaction, leading to increased entrapped air and internal voids, thereby reducing load-bearing capacity^106^. In both cases, the highest strength is achieved when there is enough binder paste to create a dense microstructure and a thin, well-bonded ITZ around aggregates, and any further increase in aggregate disrupts that balance^103^. Hence, optimal coarse aggregate content is critical for maintaining structural integrity and strength.

### Individual conditional expectation

The Individual Conditional Expectation (ICE) plots provide a more detailed perspective on how each feature influences compressive strength at an individual data point level. These plots visualize the variation in predictions across different feature values while maintaining interactions with other variables^107^.

**Fig. 19:**
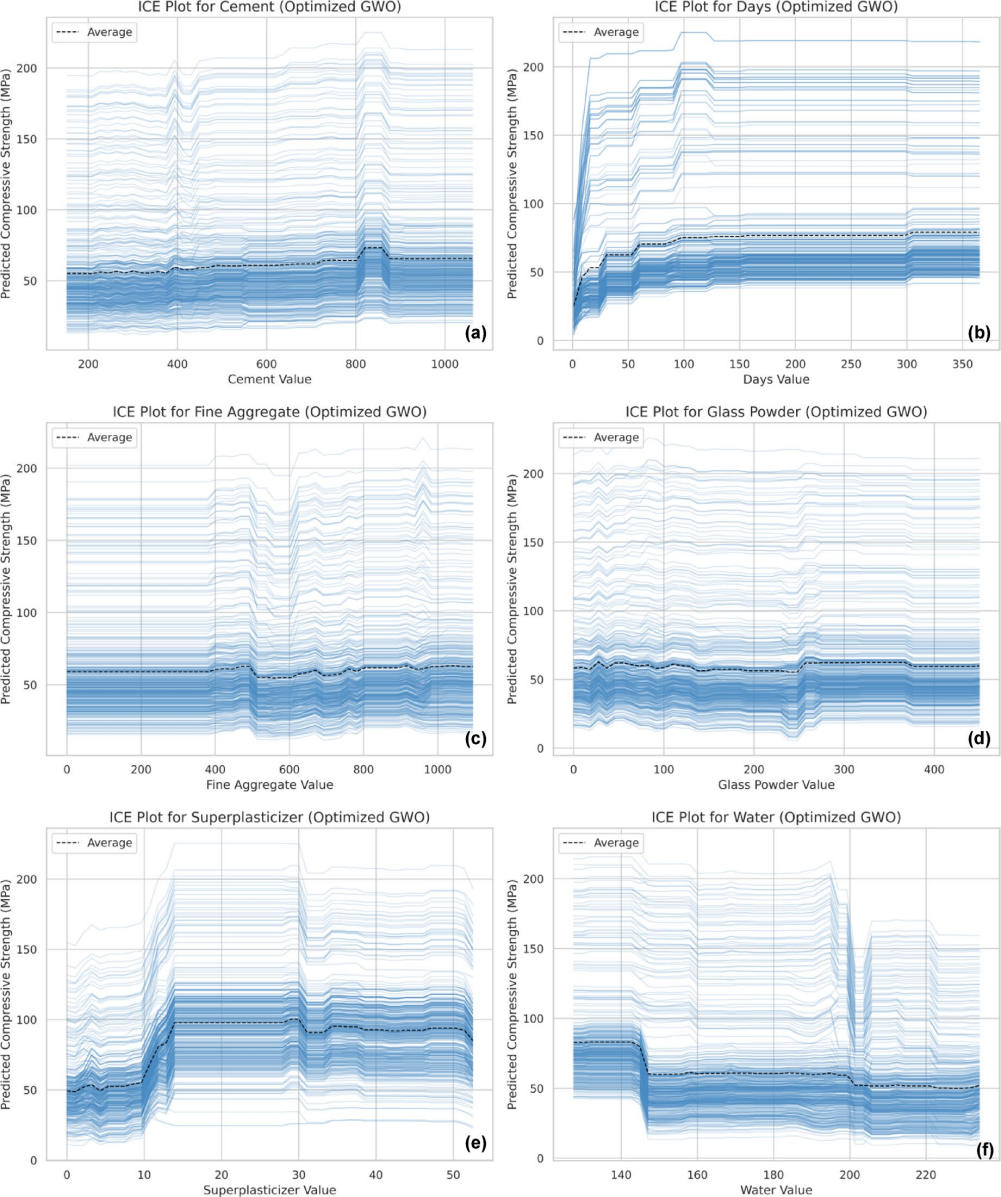

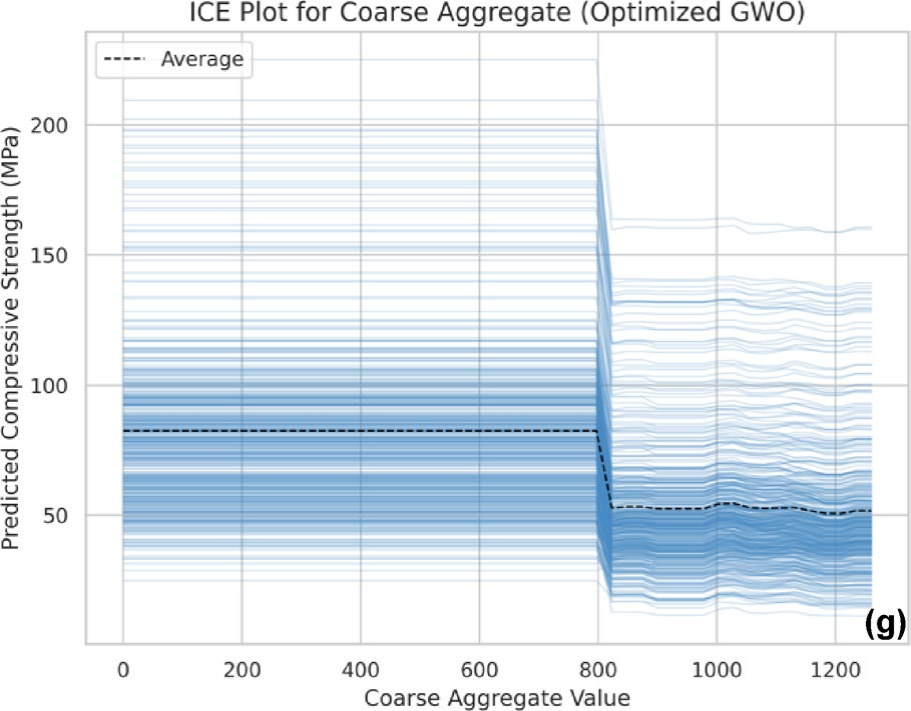
Individual Conditional Expectation (ICE) plots illustrating the effect of varying individual input features on compressive strength predictions, (**a**) Cement, (**b**) Curing Days, (**c**) Fine Aggregate, (**d**) Glass Powder, (**e**) Superplasticizer, (**f**) Water, and (**g**) Coarse Aggregate.

The ICE analysis further reinforces the trends observed in SHAP and PDP, confirming the significant roles of coarse aggregate, curing days, and superplasticizer dosage in predicting compressive strength (Fig. 19(a-g)). In the ICE plots, the black dashed line represents the average trend across all instances, offering a generalized view of how a feature impacts the output on average. The thin blue lines depict individual sample trajectories, illustrating how compressive strength predictions change as each sample’s feature value varies while keeping other variables fixed.

The ICE plot for cement (Fig. 19a) shows a predominantly increasing trend, indicating a positive effect on CS, though it stabilizes at higher values, suggesting a saturation point. The curing days plot (Fig. 19b) confirms a strong positive correlation, with strength increasing significantly within the early curing period before reaching a plateau. The fine aggregate (Fig. 19c) and glass powder (Fig. 19d) plots exhibit minimal variation, confirming their relatively lower influence on strength development. The superplasticizer plot (Fig. 19e) highlights an optimal dosage range, beyond which no significant improvement is observed. The water content plot (Fig. 19f) aligns with previous findings, where excessive water reduces strength due to increased porosity. Lastly, the coarse aggregate ICE plot (Fig. 19g) underscores its diminishing effect at higher levels, supporting earlier observations that excessive usage may disrupt the concrete matrix.

## Future perspectives

The results of this research demonstrate how well ML predicts HSGPC’s CS. Nonetheless, there are still a few crucial areas for further study and advancement:

- Incorporating detailed particle size distribution data for glass powder could refine the model’s predictive accuracy and provide deeper insights into its role in cementitious reactions.
- Exploring deep learning architectures such as transformers, graph neural networks (GNNs), and attention-based models may further enhance predictive performance and generalization.
- Increasing the dataset with a wider variety of mix compositions, curing conditions, and environmental exposure scenarios would improve the robustness of the models.
- Future developments should include real-time mix optimization, predictive analytics for durability performance, and cloud-based computing integration to make the tool more interactive and practical for users.
- Sustainability Analysis: Extending the model to evaluate the environmental impact of different mix compositions by integrating life-cycle assessment (LCA) tools could support sustainable concrete mix designs.

By addressing these future directions, ML-based predictive tools can evolve into more precise, scalable, and user-friendly systems, ultimately benefiting researchers, engineers, and the construction industry.

### Research limitations

While this study successfully demonstrates the utilization of ML in predicting the CS of high-strength concrete incorporating glass powder, certain limitations must be acknowledged. Achieving HSC is not solely dependent on glass powder; rather, it results from the combined effects of various SCMs. However, due to data limitations and the inherent variability in experimental datasets, this research focuses specifically on the role of glass powder alongside superplasticizers in enhancing concrete performance. The exclusion of other SCMs, such as fly ash, silica fume, or metakaolin, may limit the model’s ability to generalize across a broader range of high-performance mix designs. Future studies should incorporate a more comprehensive dataset encompassing multiple SCMs to refine predictive accuracy and provide a more complete understanding of their synergistic effects in achieving high-strength concrete.

## Conclusion

This research was conducted to optimize machine learning models for forecasting the CS of HSGPC. Predictive models like KNN, RF, and XGB were investigated in the study, and nature-inspired optimization approaches (PSO, FA, and GWO) were used to further improve them. To evaluate the model predictions and comprehend the impact of important input factors, SHAP, PDP, and ICE analyses were performed. The key findings are summarized as follows:

- XGB and RF outperformed KNN and RF in terms of predictive accuracy among standalone models, obtaining R^2^ values on the test dataset of 0.946 and 0.963, respectively.
- Based on the comparative analysis of standalone and optimized models, XGB-GWO exhibited superior predictive capability, attaining the highest R² (0.991) and the least MSE (14.42), demonstrating the effectiveness of hyperparameter tuning through metaheuristic algorithms.
- Taylor diagram analysis provided a compact visual validation of all models by jointly comparing their correlation, standard deviation, and RMSE. The XGB-GWO model consistently plotted nearest to the reference point in both training and testing phases, confirming its superior accuracy and generalization performance over the other models.
- SHAP analysis revealed that coarse aggregate (SHAP impact = 8.46), with excessive content leading to strength reduction due to weaker paste-aggregate bonding. Curing days (SHAP = 10.49) strongly influenced strength development, confirming that prolonged curing enhances strength.
- Superplasticizer dosage (SHAP = 11.65) improved strength within an optimal range but plateaued at higher concentrations. Water content (SHAP = 4.42) exhibited a negative impact beyond optimal levels due to increased porosity, leading to strength reduction.
- Dependence plots (PDP and ICE) further validated these findings, highlighting non-linear trends. Cement content exhibited a positive correlation with strength, but beyond ~ 600 kg/m³, the marginal gain decreased due to hydration inefficiencies. Coarse aggregate showed a strength decline beyond ~ 800 kg/m³, reinforcing the trade-off between particle packing and paste bonding.
- Although the study highlights the potential of ML in concrete mix design optimization, larger datasets, additional SCMs, and more sophisticated hybrid models should be incorporated into future studies to achieve even greater gains.

## **Declaration of Generative AI and AI-assisted technologies in the writing process**

During the preparation of this work the authors used ChatGPT only to improve readability and language of the work. After using this tool, the authors reviewed and edited the content as needed under strict human oversight and take full responsibility for the content of the publication.

## Electronic supplementary material

Below is the link to the electronic supplementary material.

Supplementary Material 1

## Data Availability

The data is available from the corresponding author upon request.
