# Supplementary material for: Machine learning prediction and explainability analysis of high strength glass powder concrete using SHAP PDP and ICE
Source: Sci Rep. 2025 Jul 1;15:22089. doi: 10.1038/s41598-025-04762-2 (PMC12219312; doi:10.1038/s41598-025-04762-2)
Supplement: Supplementary file 1 — Supplementary Material 1 [file 41598_2025_4762_MOESM1_ESM.docx]

**AI-Optimized Prediction and Explainability of High-Strength Glass Powder Concrete: SHAP, PDP, and ICE analysis**

**Supplementary File**

1. **Data Source References with Access Links**

This provides a detailed list of the original dataset sources used in this study. Each reference includes a direct link to publicly available data for easy access.

1. S.C. Kou, F. Xing [41]. Available at: <https://doi.org/10.1155/2012/263243>
2. J. Ahmad, Z. Zhou [42]. Available at: <https://doi.org/10.1016/j.conbuildmat.2023.133760>
3. M. Chandra Sekhar et. al. [43]. Available at: <https://doi.org/10.1016/j.matpr.2023.03.713>
4. T.S. Mustafa et. al. [44]. Available at: <https://doi.org/10.1016/j.istruc.2023.05.101>
5. L. Peng et. al. [45]. Available at: <https://doi.org/10.1016/J.CEMCONCOMP.2022.104909>
6. H. Du, K.H. Tan [1]. Available at: <https://doi.org/10.1016/J.CEMCONCOMP.2016.10.010>
7. K.I.M. Ibrahim [46]. Available at: <https://doi.org/10.1016/J.CSCM.2021.E00630>
8. A.T. Azeez et. al. [47]. Available at: <https://doi.org/10.1016/J.CONBUILDMAT.2023.134199>
9. A.M. Tahwia et. al. [48]. Available at: <https://doi.org/10.1016/J.CSCM.2022.E01648>
10. A.H. Alateah et. al. [49]. Available at: <https://doi.org/10.1016/J.JMRT.2022.11.180>
11. Zidol et. al. [50]. Available at: <https://doi.org/10.4236/NJGC.2017.72004>.
12. D. Paul et. al. [51]. Available at: <https://doi.org/10.1016/J.CONBUILDMAT.2022.129217>
13. N.A. Soliman et. al. [52]. Available at: <https://doi.org/10.1016/J.CONBUILDMAT.2016.08.073>
14. J. Gupta et. al. [53]. Available at: <https://doi.org/10.1016/J.MATPR.2021.09.446>
15. A.M. Tahwia et. al. [54]. Available at: <https://doi.org/10.1016/J.CONBUILDMAT.2024.136422>
16. D.A. Muhedin et. al. [55]. Available at: <https://doi.org/10.1016/J.CSCM.2023.E02512>
17. P. min Zhan et. al. [56]. Available at: <https://doi.org/10.1016/J.JCLEPRO.2022.130892>
18. H. Du et. al. [57]. Available at: <https://doi.org/10.3151/JACT.12.468>
19. J. Xu et. al. [58]. Available at: <https://doi.org/10.1016/J.POWTEC.2023.118356>
20. M.H. Lai et. al. [59]. Available at: <https://doi.org/10.1016/J.JOBE.2024.108758>
21. M. Orouji et. al. [60]. Available at: <https://doi.org/10.1016/J.ISTRUC.2021.07.048>
22. R.U.D. Nassar et. al. [61]. Available at: <https://doi.org/10.1016/J.CSCM.2021.E00745>
23. A.A. Aliabdo et. al. [62]. Available at: <https://doi.org/10.1016/J.CONBUILDMAT.2016.08.016>
24. F. Althoey et. al. [63]. Available at: <https://doi.org/10.1016/J.ASEJ.2023.102247>
25. S.H. Chu et. al. [64]. Available at: <https://doi.org/10.1016/J.CONBUILDMAT.2021.125719>
26. S. Saify et. al. [65]. Available at: <https://doi.org/10.1515/eng-2022-0479>

**S2. Dataset:**

| **Sr. #** | **C** | **GP** | **FA** | **CA** | **W** | **SP** | **D** | **CS** |
| --- | --- | --- | --- | --- | --- | --- | --- | --- |
|  | *Kg/m^3^* | *Kg/m^3^* | *Kg/m^3^* | *Kg/m^3^* | *Kg/m^3^* | *Kg/m^3^* | *days* | *MPa* |
| 1 | 1062 | 0 | 1094 | 0 | 159.3 | 11.15 | 1 | 96 |
|  | 903 | 159 | 1058 | 0 | 159.3 | 11.15 | 1 | 89 |
|  | 743 | 319 | 1021 | 0 | 159.3 | 11.15 | 1 | 85 |
|  | 1062 | 0 | 1094 | 0 | 159.3 | 11.15 | 28 | 143 |
|  | 903 | 159 | 1058 | 0 | 159.3 | 11.15 | 28 | 147 |
|  | 743 | 319 | 1021 | 0 | 159.3 | 11.15 | 28 | 152 |
|  | 1062 | 0 | 1094 | 0 | 159.3 | 11.15 | 90 | 147 |
|  | 903 | 159 | 1058 | 0 | 159.3 | 11.15 | 90 | 153 |
|  | 743 | 319 | 1021 | 0 | 159.3 | 11.15 | 90 | 159 |
|  | 1062 | 0 | 1094 | 0 | 159.3 | 11.15 | 365 | 158 |
|  | 903 | 159 | 1058 | 0 | 159.3 | 11.15 | 365 | 162 |
|  | 743 | 319 | 1021 | 0 | 159.3 | 11.15 | 365 | 168 |
| 2 | 420 | 0 | 567 | 1260 | 231 | 5.9 | 7 | 16 |
|  | 399 | 21 | 567 | 1260 | 231 | 5.9 | 7 | 17.5 |
|  | 378 | 42 | 567 | 1260 | 231 | 5.9 | 7 | 18.1 |
|  | 357 | 63 | 567 | 1260 | 231 | 5.9 | 7 | 18.3 |
|  | 336 | 84 | 567 | 1260 | 231 | 5.9 | 7 | 16.1 |
|  | 420 | 0 | 567 | 1260 | 231 | 5.9 | 28 | 39 |
|  | 399 | 21 | 567 | 1260 | 231 | 5.9 | 28 | 43.3 |
|  | 378 | 42 | 567 | 1260 | 231 | 5.9 | 28 | 46.3 |
|  | 357 | 63 | 567 | 1260 | 231 | 5.9 | 28 | 45.8 |
|  | 336 | 84 | 567 | 1260 | 231 | 5.9 | 28 | 41.3 |
|  | 420 | 0 | 567 | 1260 | 231 | 5.9 | 56 | 41.1 |
|  | 399 | 21 | 567 | 1260 | 231 | 5.9 | 56 | 44.5 |
|  | 378 | 42 | 567 | 1260 | 231 | 5.9 | 56 | 49.2 |
|  | 357 | 63 | 567 | 1260 | 231 | 5.9 | 56 | 48.4 |
|  | 336 | 84 | 567 | 1260 | 231 | 5.9 | 56 | 46 |
| 3 | 412 | 0 | 636 | 1221 | 167 | 3.7 | 7 | 32.3 |
|  | 412 | 31.8 | 604 | 1221 | 167 | 3.7 | 7 | 33.8 |
|  | 412 | 63.6 | 572 | 1221 | 167 | 3.7 | 7 | 34.7 |
|  | 412 | 95.4 | 540 | 1221 | 167 | 3.7 | 7 | 33.2 |
|  | 412 | 0 | 636 | 1221 | 167 | 3.7 | 28 | 49.2 |
|  | 412 | 31.8 | 604 | 1221 | 167 | 3.7 | 28 | 49.4 |
|  | 412 | 63.6 | 572 | 1221 | 167 | 3.7 | 28 | 51.3 |
|  | 412 | 95.4 | 540 | 1221 | 167 | 3.7 | 28 | 50.7 |
| 4 | 350 | 0 | 720 | 1200 | 175 | 3.5 | 7 | 31 |
|  | 315 | 35 | 720 | 1200 | 175 | 3.5 | 7 | 32.5 |
|  | 280 | 70 | 720 | 1200 | 175 | 3.5 | 7 | 28.8 |
|  | 350 | 0 | 720 | 1200 | 175 | 3.5 | 28 | 40 |
|  | 315 | 35 | 720 | 1200 | 175 | 3.5 | 28 | 41.7 |
|  | 280 | 70 | 720 | 1200 | 175 | 3.5 | 28 | 38.4 |
| 5 | 345 | 0 | 766 | 1149 | 228.6 | 0 | 3 | 26.3 |
|  | 310.5 | 34.5 | 766 | 1149 | 228.6 | 0 | 3 | 23.1 |
|  | 276 | 69 | 766 | 1149 | 228.6 | 0 | 3 | 21.8 |
|  | 241.5 | 103.5 | 766 | 1149 | 228.6 | 0 | 3 | 19.1 |
|  | 207 | 138 | 766 | 1149 | 228.6 | 0 | 3 | 16.1 |
|  | 345 | 0 | 766 | 1149 | 228.6 | 0 | 28 | 38.6 |
|  | 310.5 | 34.5 | 766 | 1149 | 228.6 | 0 | 28 | 36.3 |
|  | 276 | 69 | 766 | 1149 | 228.6 | 0 | 28 | 36.8 |
|  | 241.5 | 103.5 | 766 | 1149 | 228.6 | 0 | 28 | 28.4 |
|  | 207 | 138 | 766 | 1149 | 228.6 | 0 | 28 | 26.1 |
|  | 345 | 0 | 766 | 1149 | 228.6 | 0 | 90 | 46 |
|  | 310.5 | 34.5 | 766 | 1149 | 228.6 | 0 | 90 | 47.5 |
|  | 276 | 69 | 766 | 1149 | 228.6 | 0 | 90 | 48.3 |
|  | 241.5 | 103.5 | 766 | 1149 | 228.6 | 0 | 90 | 38.6 |
|  | 207 | 138 | 766 | 1149 | 228.6 | 0 | 90 | 37.1 |
| 6 | 380 | 0 | 960 | 825 | 185 | 3.8 | 3 | 36.5 |
|  | 323 | 57 | 960 | 825 | 185 | 3.2 | 3 | 35 |
|  | 266 | 114 | 960 | 825 | 185 | 3 | 3 | 31 |
|  | 209 | 171 | 960 | 825 | 185 | 3.9 | 3 | 28 |
|  | 152 | 228 | 960 | 825 | 185 | 4.5 | 3 | 22 |
|  | 380 | 0 | 960 | 825 | 185 | 3.8 | 28 | 42.2 |
|  | 323 | 57 | 960 | 825 | 185 | 3.2 | 28 | 45 |
|  | 266 | 114 | 960 | 825 | 185 | 3 | 28 | 46.5 |
|  | 209 | 171 | 960 | 825 | 185 | 3.9 | 28 | 45.4 |
|  | 152 | 228 | 960 | 825 | 185 | 4.5 | 28 | 39 |
|  | 380 | 0 | 960 | 825 | 185 | 3.8 | 365 | 48.1 |
|  | 323 | 57 | 960 | 825 | 185 | 3.2 | 365 | 63.2 |
|  | 266 | 114 | 960 | 825 | 185 | 3 | 365 | 61.5 |
|  | 209 | 171 | 960 | 825 | 185 | 3.9 | 365 | 58.1 |
|  | 152 | 228 | 960 | 825 | 185 | 4.5 | 365 | 55.3 |
| 7 | 400 | 0 | 594 | 0 | 200 | 4 | 28 | 33.5 |
|  | 380 | 20 | 594 | 0 | 200 | 4 | 28 | 36 |
|  | 360 | 40 | 594 | 0 | 200 | 4 | 28 | 32.3 |
|  | 340 | 60 | 594 | 0 | 200 | 4 | 28 | 31.2 |
|  | 320 | 80 | 594 | 0 | 200 | 4 | 28 | 26.2 |
|  | 500 | 0 | 566 | 0 | 200 | 12.5 | 28 | 48.5 |
|  | 475 | 25 | 566 | 0 | 200 | 12.5 | 28 | 47.4 |
|  | 450 | 50 | 566 | 0 | 200 | 12.5 | 28 | 44.6 |
|  | 425 | 75 | 566 | 0 | 200 | 12.5 | 28 | 44.1 |
|  | 400 | 100 | 566 | 0 | 200 | 12.5 | 28 | 42.1 |
|  | 500 | 0 | 566 | 0 | 200 | 12 | 28 | 52.5 |
|  | 475 | 25 | 566 | 0 | 200 | 12 | 28 | 49.8 |
|  | 450 | 50 | 566 | 0 | 200 | 12 | 28 | 48.06 |
|  | 425 | 75 | 566 | 0 | 200 | 12 | 28 | 47.2 |
|  | 400 | 100 | 566 | 0 | 200 | 12 | 28 | 45.1 |
| 8 | 425 | 0 | 750 | 1020 | 170 | 2.12 | 28 | 55.55 |
|  | 327.2 | 97.75 | 750 | 1020 | 195.5 | 0.85 | 28 | 41.3 |
|  | 361.2 | 63.75 | 750 | 1020 | 170 | 2.12 | 28 | 56.17 |
|  | 361.2 | 63.75 | 750 | 1020 | 170 | 2.12 | 28 | 57.45 |
|  | 395.25 | 29.75 | 750 | 1020 | 195.5 | 0.85 | 28 | 52.79 |
|  | 327.2 | 97.75 | 750 | 1020 | 195.5 | 0.85 | 28 | 48.86 |
|  | 395.25 | 29.75 | 750 | 1020 | 195.5 | 0.85 | 28 | 57.65 |
|  | 327.2 | 97.75 | 750 | 1020 | 144.5 | 2.9 | 28 | 62.68 |
|  | 395.25 | 29.75 | 750 | 1020 | 144.5 | 2.9 | 28 | 72.94 |
|  | 327.2 | 97.75 | 750 | 1020 | 144.5 | 2.9 | 28 | 78.09 |
|  | 395.25 | 29.75 | 750 | 1020 | 144.5 | 2.12 | 28 | 79.75 |
|  | 361.2 | 63.75 | 750 | 1020 | 170 | 2.12 | 28 | 53.82 |
|  | 361.2 | 63.75 | 750 | 1020 | 170 | 2.12 | 28 | 61.48 |
|  | 361.2 | 127.5 | 750 | 1020 | 170 | 2.12 | 28 | 48.1 |
|  | 361.2 | 63.75 | 750 | 1020 | 170 | 2.12 | 28 | 45.35 |
|  | 361.2 | 63.75 | 750 | 1020 | 170 | 2.12 | 28 | 44.64 |
|  | 425 | 0 | 750 | 1020 | 170 | 2.12 | 28 | 54.6 |
|  | 361.2 | 63.75 | 750 | 1020 | 170 | 2.12 | 28 | 54.29 |
|  | 361.2 | 63.75 | 750 | 1020 | 127.5 | 5.1 | 28 | 82.81 |
|  | 425 | 0 | 750 | 1020 | 170 | 2.12 | 90 | 59.14 |
|  | 327.2 | 97.75 | 750 | 1020 | 195.5 | 0.85 | 90 | 46.6 |
|  | 361.2 | 63.75 | 750 | 1020 | 170 | 2.12 | 90 | 68.79 |
|  | 361.2 | 63.75 | 750 | 1020 | 170 | 2.12 | 90 | 67.28 |
|  | 395.25 | 29.75 | 750 | 1020 | 195.5 | 0.85 | 90 | 63.4 |
|  | 327.2 | 97.75 | 750 | 1020 | 195.5 | 0.85 | 90 | 58.19 |
|  | 395.25 | 29.75 | 750 | 1020 | 195.5 | 0.85 | 90 | 65.94 |
|  | 327.2 | 97.75 | 750 | 1020 | 144.5 | 2.9 | 90 | 75.87 |
|  | 395.25 | 29.75 | 750 | 1020 | 144.5 | 2.9 | 90 | 80.68 |
|  | 327.2 | 97.75 | 750 | 1020 | 144.5 | 2.9 | 90 | 93.53 |
|  | 395.25 | 29.75 | 750 | 1020 | 144.5 | 2.12 | 90 | 88.28 |
|  | 361.2 | 63.75 | 750 | 1020 | 170 | 2.12 | 90 | 64.57 |
|  | 361.2 | 63.75 | 750 | 1020 | 170 | 2.12 | 90 | 70.98 |
|  | 361.2 | 127.5 | 750 | 1020 | 170 | 2.12 | 90 | 59.53 |
|  | 361.2 | 63.75 | 750 | 1020 | 170 | 2.12 | 90 | 54.54 |
|  | 361.2 | 63.75 | 750 | 1020 | 170 | 2.12 | 90 | 48.46 |
|  | 425 | 0 | 750 | 1020 | 170 | 2.12 | 90 | 65.51 |
|  | 361.2 | 63.75 | 750 | 1020 | 170 | 2.12 | 90 | 64.93 |
|  | 361.2 | 63.75 | 750 | 1020 | 127.5 | 5.1 | 90 | 87.14 |
| 9 | 900 | 0 | 950 | 0 | 144 | 28 | 1 | 60 |
|  | 810 | 90 | 950 | 0 | 144 | 28 | 1 | 65 |
|  | 630 | 270 | 950 | 0 | 144 | 28 | 1 | 48 |
|  | 450 | 450 | 950 | 0 | 144 | 28 | 1 | 43 |
|  | 900 | 0 | 950 | 0 | 144 | 28 | 7 | 140 |
|  | 810 | 90 | 950 | 0 | 144 | 28 | 7 | 152 |
|  | 630 | 270 | 950 | 0 | 144 | 28 | 7 | 135 |
|  | 450 | 450 | 950 | 0 | 144 | 28 | 7 | 127 |
|  | 900 | 0 | 950 | 0 | 144 | 28 | 28 | 181 |
|  | 810 | 90 | 950 | 0 | 144 | 28 | 28 | 212 |
|  | 630 | 270 | 950 | 0 | 144 | 28 | 28 | 175 |
|  | 450 | 450 | 950 | 0 | 144 | 28 | 28 | 171 |
|  | 900 | 0 | 950 | 0 | 144 | 28 | 56 | 192 |
|  | 810 | 90 | 950 | 0 | 144 | 28 | 56 | 219 |
|  | 630 | 270 | 950 | 0 | 144 | 28 | 56 | 189 |
|  | 450 | 450 | 950 | 0 | 144 | 28 | 56 | 184 |
|  | 900 | 0 | 950 | 0 | 144 | 28 | 91 | 216 |
|  | 810 | 90 | 950 | 0 | 144 | 28 | 91 | 225 |
|  | 630 | 270 | 950 | 0 | 144 | 28 | 91 | 198 |
|  | 450 | 450 | 950 | 0 | 144 | 28 | 91 | 191 |
| 10 | 350 | 0 | 798 | 1093 | 147 | 1.75 | 3 | 30.2 |
|  | 315 | 0 | 786 | 1093 | 147 | 1.75 | 3 | 29.3 |
|  | 315 | 8.75 | 787 | 1093 | 147 | 1.75 | 3 | 26.55 |
|  | 315 | 17.5 | 788 | 1093 | 147 | 1.75 | 3 | 24 |
|  | 315 | 26.25 | 789 | 1093 | 147 | 1.75 | 3 | 27.29 |
|  | 315 | 35 | 790 | 1093 | 147 | 1.75 | 3 | 19 |
|  | 350 | 0 | 798 | 1093 | 147 | 1.75 | 7 | 37.26 |
|  | 315 | 0 | 786 | 1093 | 147 | 1.75 | 7 | 37.61 |
|  | 315 | 8.75 | 787 | 1093 | 147 | 1.75 | 7 | 35.36 |
|  | 315 | 17.5 | 788 | 1093 | 147 | 1.75 | 7 | 28.15 |
|  | 315 | 26.25 | 789 | 1093 | 147 | 1.75 | 7 | 37.05 |
|  | 315 | 35 | 790 | 1093 | 147 | 1.75 | 7 | 24.6 |
|  | 350 | 0 | 798 | 1093 | 147 | 1.75 | 14 | 41.3 |
|  | 315 | 0 | 786 | 1093 | 147 | 1.75 | 14 | 41 |
|  | 315 | 8.75 | 787 | 1093 | 147 | 1.75 | 14 | 40.28 |
|  | 315 | 17.5 | 788 | 1093 | 147 | 1.75 | 14 | 34 |
|  | 315 | 26.25 | 789 | 1093 | 147 | 1.75 | 14 | 41.7 |
|  | 315 | 35 | 790 | 1093 | 147 | 1.75 | 14 | 26.9 |
|  | 350 | 0 | 798 | 1093 | 147 | 1.75 | 28 | 49 |
|  | 315 | 0 | 786 | 1093 | 147 | 1.75 | 28 | 46 |
|  | 315 | 8.75 | 787 | 1093 | 147 | 1.75 | 28 | 47.4 |
|  | 315 | 17.5 | 788 | 1093 | 147 | 1.75 | 28 | 36.9 |
|  | 315 | 26.25 | 789 | 1093 | 147 | 1.75 | 28 | 45.9 |
|  | 315 | 35 | 790 | 1093 | 147 | 1.75 | 28 | 31 |
|  | 350 | 0 | 798 | 1093 | 147 | 1.75 | 56 | 52 |
|  | 315 | 0 | 786 | 1093 | 147 | 1.75 | 56 | 49 |
|  | 315 | 8.75 | 787 | 1093 | 147 | 1.75 | 56 | 50.5 |
|  | 315 | 17.5 | 788 | 1093 | 147 | 1.75 | 56 | 40 |
|  | 315 | 26.25 | 789 | 1093 | 147 | 1.75 | 56 | 49.1 |
|  | 315 | 35 | 790 | 1093 | 147 | 1.75 | 56 | 33 |
|  | 350 | 0 | 798 | 1093 | 147 | 1.75 | 90 | 54 |
|  | 315 | 0 | 786 | 1093 | 147 | 1.75 | 90 | 50.3 |
|  | 315 | 8.75 | 787 | 1093 | 147 | 1.75 | 90 | 55.5 |
|  | 315 | 17.5 | 788 | 1093 | 147 | 1.75 | 90 | 47.9 |
|  | 315 | 26.25 | 789 | 1093 | 147 | 1.75 | 90 | 56.4 |
|  | 315 | 35 | 790 | 1093 | 147 | 1.75 | 90 | 37 |
| 11 | 400 | 0 | 690 | 1070 | 160 | 9.32 | 1 | 29 |
|  | 320 | 80 | 673 | 1070 | 160 | 8.12 | 1 | 23 |
|  | 280 | 120 | 664 | 1070 | 160 | 8 | 1 | 17 |
|  | 400 | 0 | 690 | 1070 | 160 | 9.32 | 28 | 49 |
|  | 320 | 80 | 673 | 1070 | 160 | 8.12 | 28 | 44 |
|  | 280 | 120 | 664 | 1070 | 160 | 8 | 28 | 39 |
|  | 400 | 0 | 690 | 1070 | 160 | 9.32 | 56 | 53 |
|  | 320 | 80 | 673 | 1070 | 160 | 8.12 | 56 | 50 |
|  | 280 | 120 | 664 | 1070 | 160 | 8 | 56 | 47 |
|  | 400 | 0 | 690 | 1070 | 160 | 9.32 | 91 | 58 |
|  | 320 | 80 | 673 | 1070 | 160 | 8.12 | 91 | 54 |
|  | 280 | 120 | 664 | 1070 | 160 | 8 | 91 | 52 |
|  | 400 | 0 | 690 | 1070 | 160 | 9.32 | 365 | 59 |
|  | 320 | 80 | 673 | 1070 | 160 | 8.12 | 365 | 64 |
|  | 280 | 120 | 664 | 1070 | 160 | 8 | 365 | 58 |
|  | 430 | 0 | 691 | 1070 | 150.5 | 11.48 | 1 | 29 |
|  | 344 | 86 | 672 | 1070 | 150.5 | 9.46 | 1 | 27 |
|  | 301 | 129 | 662 | 1070 | 150.5 | 7.65 | 1 | 18 |
|  | 430 | 0 | 691 | 1070 | 150.5 | 11.48 | 28 | 64 |
|  | 344 | 86 | 672 | 1070 | 150.5 | 9.46 | 28 | 54 |
|  | 301 | 129 | 662 | 1070 | 150.5 | 7.65 | 28 | 46 |
|  | 430 | 0 | 691 | 1070 | 150.5 | 11.48 | 56 | 68 |
|  | 344 | 86 | 672 | 1070 | 150.5 | 9.46 | 56 | 58 |
|  | 301 | 129 | 662 | 1070 | 150.5 | 7.65 | 56 | 52 |
|  | 430 | 0 | 691 | 1070 | 150.5 | 11.48 | 91 | 70 |
|  | 344 | 86 | 672 | 1070 | 150.5 | 9.46 | 91 | 62 |
|  | 301 | 129 | 662 | 1070 | 150.5 | 7.65 | 91 | 56 |
|  | 430 | 0 | 691 | 1070 | 150.5 | 11.48 | 365 | 78 |
|  | 344 | 86 | 672 | 1070 | 150.5 | 9.46 | 365 | 73 |
|  | 301 | 129 | 662 | 1070 | 150.5 | 7.65 | 365 | 69 |
| 12 | 319 | 0 | 697.3 | 1171.5 | 153.3 | 1.6 | 7 | 27 |
|  | 287 | 31.94 | 697.3 | 1171.5 | 153.3 | 1.6 | 7 | 26 |
|  | 255 | 63.84 | 697.3 | 1171.5 | 153.3 | 1.6 | 7 | 28 |
|  | 223.5 | 95.8 | 697.3 | 1171.5 | 153.3 | 1.6 | 7 | 29 |
|  | 191.6 | 127.7 | 697.3 | 1171.5 | 153.3 | 1.6 | 7 | 28 |
|  | 319 | 0 | 697.3 | 1171.5 | 153.3 | 1.6 | 28 | 37 |
|  | 287 | 31.94 | 697.3 | 1171.5 | 153.3 | 1.6 | 28 | 38 |
|  | 255 | 63.84 | 697.3 | 1171.5 | 153.3 | 1.6 | 28 | 39 |
|  | 223.5 | 95.8 | 697.3 | 1171.5 | 153.3 | 1.6 | 28 | 40 |
|  | 191.6 | 127.7 | 697.3 | 1171.5 | 153.3 | 1.6 | 28 | 39 |
|  | 319 | 0 | 697.3 | 1171.5 | 153.3 | 1.6 | 90 | 38 |
|  | 287 | 31.94 | 697.3 | 1171.5 | 153.3 | 1.6 | 90 | 39 |
|  | 255 | 63.84 | 697.3 | 1171.5 | 153.3 | 1.6 | 90 | 40 |
|  | 223.5 | 95.8 | 697.3 | 1171.5 | 153.3 | 1.6 | 90 | 42 |
|  | 191.6 | 127.7 | 697.3 | 1171.5 | 153.3 | 1.6 | 90 | 40 |
|  | 368 | 0 | 663.7 | 1163.4 | 147.5 | 1.84 | 7 | 28.5 |
|  | 331.9 | 36.88 | 663.7 | 1163.4 | 147.5 | 1.84 | 7 | 27 |
|  | 295.04 | 73.76 | 663.7 | 1163.4 | 147.5 | 1.84 | 7 | 29.5 |
|  | 258.16 | 110.6 | 663.7 | 1163.4 | 147.5 | 1.84 | 7 | 30 |
|  | 221.28 | 147.52 | 663.7 | 1163.4 | 147.5 | 1.84 | 7 | 28 |
|  | 368 | 0 | 663.7 | 1163.4 | 147.5 | 1.84 | 28 | 36 |
|  | 331.9 | 36.88 | 663.7 | 1163.4 | 147.5 | 1.84 | 28 | 37 |
|  | 295.04 | 73.76 | 663.7 | 1163.4 | 147.5 | 1.84 | 28 | 38 |
|  | 258.16 | 110.6 | 663.7 | 1163.4 | 147.5 | 1.84 | 28 | 41 |
|  | 221.28 | 147.52 | 663.7 | 1163.4 | 147.5 | 1.84 | 28 | 38 |
|  | 368 | 0 | 663.7 | 1163.4 | 147.5 | 1.84 | 90 | 38 |
|  | 331.9 | 36.88 | 663.7 | 1163.4 | 147.5 | 1.84 | 90 | 39 |
|  | 295.04 | 73.76 | 663.7 | 1163.4 | 147.5 | 1.84 | 90 | 42 |
|  | 258.16 | 110.6 | 663.7 | 1163.4 | 147.5 | 1.84 | 90 | 44 |
|  | 221.28 | 147.52 | 663.7 | 1163.4 | 147.5 | 1.84 | 90 | 39 |
| 13 | 807 | 0 | 972 | 0 | 195 | 13 | 1 | 55 |
|  | 724 | 81 | 966 | 0 | 195 | 13 | 1 | 50 |
|  | 639 | 160 | 960 | 0 | 193 | 13 | 1 | 43 |
|  | 556 | 238 | 953 | 0 | 192 | 13 | 1 | 35 |
|  | 473 | 237 | 947 | 0 | 191 | 13 | 1 | 30 |
|  | 392 | 235 | 941 | 0 | 190 | 13 | 1 | 25 |
|  | 807 | 0 | 972 | 0 | 195 | 13 | 7 | 121 |
|  | 724 | 81 | 966 | 0 | 195 | 13 | 7 | 110 |
|  | 639 | 160 | 960 | 0 | 193 | 13 | 7 | 105 |
|  | 556 | 238 | 953 | 0 | 192 | 13 | 7 | 99 |
|  | 473 | 237 | 947 | 0 | 191 | 13 | 7 | 94 |
|  | 392 | 235 | 941 | 0 | 190 | 13 | 7 | 87 |
|  | 807 | 0 | 972 | 0 | 195 | 13 | 28 | 170 |
|  | 724 | 81 | 966 | 0 | 195 | 13 | 28 | 167 |
|  | 639 | 160 | 960 | 0 | 193 | 13 | 28 | 171 |
|  | 556 | 238 | 953 | 0 | 192 | 13 | 28 | 163 |
|  | 473 | 237 | 947 | 0 | 191 | 13 | 28 | 153 |
|  | 392 | 235 | 941 | 0 | 190 | 13 | 28 | 145 |
|  | 807 | 0 | 972 | 0 | 195 | 13 | 56 | 175 |
|  | 724 | 81 | 966 | 0 | 195 | 13 | 56 | 184 |
|  | 639 | 160 | 960 | 0 | 193 | 13 | 56 | 186 |
|  | 556 | 238 | 953 | 0 | 192 | 13 | 56 | 178 |
|  | 473 | 237 | 947 | 0 | 191 | 13 | 56 | 175 |
|  | 392 | 235 | 941 | 0 | 190 | 13 | 56 | 157 |
|  | 807 | 0 | 972 | 0 | 195 | 13 | 91 | 178 |
|  | 724 | 81 | 966 | 0 | 195 | 13 | 91 | 197 |
|  | 639 | 160 | 960 | 0 | 193 | 13 | 91 | 202 |
|  | 556 | 238 | 953 | 0 | 192 | 13 | 91 | 195 |
|  | 473 | 237 | 947 | 0 | 191 | 13 | 91 | 190 |
|  | 392 | 235 | 941 | 0 | 190 | 13 | 91 | 184 |
| 14 | 385 | 0 | 780.8 | 1069.8 | 154 | 3.08 | 28 | 38 |
|  | 365.4 | 19.25 | 780.8 | 1069.8 | 154 | 3.08 | 28 | 38.5 |
|  | 346.5 | 38.5 | 780.8 | 1069.8 | 154 | 3.08 | 28 | 39 |
|  | 327.2 | 57.75 | 780.8 | 1069.8 | 154 | 3.08 | 28 | 39.5 |
|  | 308 | 77 | 780.8 | 1069.8 | 154 | 3.08 | 28 | 41 |
|  | 288.7 | 96.25 | 780.8 | 1069.8 | 154 | 3.08 | 28 | 42 |
|  | 269.5 | 115.5 | 780.8 | 1069.8 | 154 | 3.08 | 28 | 43 |
|  | 250 | 14.75 | 780.8 | 1069.8 | 154 | 3.08 | 28 | 38 |
|  | 231 | 154 | 780.8 | 1069.8 | 154 | 3.08 | 28 | 37 |
|  | 385 | 0 | 780.8 | 1069.8 | 154 | 3.08 | 56 | 45 |
|  | 365.4 | 19.25 | 780.8 | 1069.8 | 154 | 3.08 | 56 | 46 |
|  | 346.5 | 38.5 | 780.8 | 1069.8 | 154 | 3.08 | 56 | 47 |
|  | 327.2 | 57.75 | 780.8 | 1069.8 | 154 | 3.08 | 56 | 48 |
|  | 308 | 77 | 780.8 | 1069.8 | 154 | 3.08 | 56 | 49 |
|  | 288.7 | 96.25 | 780.8 | 1069.8 | 154 | 3.08 | 56 | 49.5 |
|  | 269.5 | 115.5 | 780.8 | 1069.8 | 154 | 3.08 | 56 | 51 |
|  | 250 | 14.75 | 780.8 | 1069.8 | 154 | 3.08 | 56 | 48 |
|  | 231 | 154 | 780.8 | 1069.8 | 154 | 3.08 | 56 | 47 |
|  | 385 | 0 | 780.8 | 1069.8 | 154 | 3.08 | 90 | 52 |
|  | 365.4 | 19.25 | 780.8 | 1069.8 | 154 | 3.08 | 90 | 53 |
|  | 346.5 | 38.5 | 780.8 | 1069.8 | 154 | 3.08 | 90 | 56 |
|  | 327.2 | 57.75 | 780.8 | 1069.8 | 154 | 3.08 | 90 | 58 |
|  | 308 | 77 | 780.8 | 1069.8 | 154 | 3.08 | 90 | 62 |
|  | 288.7 | 96.25 | 780.8 | 1069.8 | 154 | 3.08 | 90 | 63 |
|  | 269.5 | 115.5 | 780.8 | 1069.8 | 154 | 3.08 | 90 | 64 |
|  | 250 | 14.75 | 780.8 | 1069.8 | 154 | 3.08 | 90 | 60 |
|  | 231 | 154 | 780.8 | 1069.8 | 154 | 3.08 | 90 | 57 |
| 15 | 480 | 0 | 647 | 1112 | 161 | 2.4 | 3 | 30 |
|  | 432 | 38 | 647 | 1112 | 161 | 2.4 | 3 | 31 |
|  | 384 | 77 | 647 | 1112 | 161 | 2.4 | 3 | 29 |
|  | 336 | 115 | 647 | 1112 | 161 | 2.4 | 3 | 25 |
|  | 288 | 154 | 647 | 1112 | 161 | 2.4 | 3 | 23 |
|  | 240 | 192 | 647 | 1112 | 161 | 2.4 | 3 | 22 |
|  | 480 | 0 | 647 | 1112 | 161 | 2.4 | 7 | 40 |
|  | 432 | 38 | 647 | 1112 | 161 | 2.4 | 7 | 42.5 |
|  | 384 | 77 | 647 | 1112 | 161 | 2.4 | 7 | 41 |
|  | 336 | 115 | 647 | 1112 | 161 | 2.4 | 7 | 34 |
|  | 288 | 154 | 647 | 1112 | 161 | 2.4 | 7 | 32 |
|  | 240 | 192 | 647 | 1112 | 161 | 2.4 | 7 | 30 |
|  | 480 | 0 | 647 | 1112 | 161 | 2.4 | 28 | 50 |
|  | 432 | 38 | 647 | 1112 | 161 | 2.4 | 28 | 52.5 |
|  | 384 | 77 | 647 | 1112 | 161 | 2.4 | 28 | 51 |
|  | 336 | 115 | 647 | 1112 | 161 | 2.4 | 28 | 40 |
|  | 288 | 154 | 647 | 1112 | 161 | 2.4 | 28 | 38 |
|  | 240 | 192 | 647 | 1112 | 161 | 2.4 | 28 | 35 |
|  | 480 | 0 | 647 | 1112 | 161 | 2.4 | 56 | 55 |
|  | 432 | 38 | 647 | 1112 | 161 | 2.4 | 56 | 58 |
|  | 384 | 77 | 647 | 1112 | 161 | 2.4 | 56 | 53 |
|  | 336 | 115 | 647 | 1112 | 161 | 2.4 | 56 | 48 |
|  | 288 | 154 | 647 | 1112 | 161 | 2.4 | 56 | 46 |
|  | 240 | 192 | 647 | 1112 | 161 | 2.4 | 56 | 40 |
| 16 | 465 | 0 | 715 | 1030 | 200 | 0 | 7 | 28 |
|  | 441.75 | 23.25 | 715 | 1030 | 200 | 0 | 7 | 37 |
|  | 418.5 | 46.5 | 715 | 1030 | 200 | 0 | 7 | 34 |
|  | 395.25 | 69.75 | 715 | 1030 | 200 | 0 | 7 | 30 |
|  | 372 | 93 | 715 | 1030 | 200 | 0 | 7 | 25 |
|  | 465 | 0 | 715 | 1030 | 200 | 0 | 28 | 42 |
|  | 441.75 | 23.25 | 715 | 1030 | 200 | 0 | 28 | 48 |
|  | 418.5 | 46.5 | 715 | 1030 | 200 | 0 | 28 | 46 |
|  | 395.25 | 69.75 | 715 | 1030 | 200 | 0 | 28 | 43 |
|  | 372 | 93 | 715 | 1030 | 200 | 0 | 28 | 36 |
|  | 465 | 0 | 715 | 1030 | 200 | 0 | 60 | 47 |
|  | 441.75 | 23.25 | 715 | 1030 | 200 | 0 | 60 | 58 |
|  | 418.5 | 46.5 | 715 | 1030 | 200 | 0 | 60 | 56 |
|  | 395.25 | 69.75 | 715 | 1030 | 200 | 0 | 60 | 50 |
|  | 372 | 93 | 715 | 1030 | 200 | 0 | 60 | 42 |
|  | 465 | 0 | 715 | 1030 | 200 | 0 | 90 | 55 |
|  | 441.75 | 23.25 | 715 | 1030 | 200 | 0 | 90 | 65 |
|  | 418.5 | 46.5 | 715 | 1030 | 200 | 0 | 90 | 61 |
|  | 395.25 | 69.75 | 715 | 1030 | 200 | 0 | 90 | 56 |
|  | 372 | 93 | 715 | 1030 | 200 | 0 | 90 | 47 |
| 17 | 450 | 0 | 713 | 1070 | 157 | 4.85 | 3 | 23 |
|  | 315 | 45 | 713 | 1070 | 157 | 4.52 | 3 | 27 |
|  | 270 | 90 | 713 | 1070 | 157 | 4.36 | 3 | 17 |
|  | 225 | 135 | 713 | 1070 | 157 | 4.28 | 3 | 18 |
|  | 315 | 90 | 713 | 1070 | 157 | 4.48 | 3 | 13 |
|  | 225 | 90 | 713 | 1070 | 157 | 4.67 | 3 | 21 |
|  | 450 | 0 | 713 | 1070 | 157 | 4.85 | 7 | 28 |
|  | 315 | 45 | 713 | 1070 | 157 | 4.52 | 7 | 40 |
|  | 270 | 90 | 713 | 1070 | 157 | 4.36 | 7 | 24 |
|  | 225 | 135 | 713 | 1070 | 157 | 4.28 | 7 | 27 |
|  | 315 | 90 | 713 | 1070 | 157 | 4.48 | 7 | 23 |
|  | 225 | 90 | 713 | 1070 | 157 | 4.67 | 7 | 33 |
|  | 450 | 0 | 713 | 1070 | 157 | 4.85 | 28 | 36.5 |
|  | 315 | 45 | 713 | 1070 | 157 | 4.52 | 28 | 47 |
|  | 270 | 90 | 713 | 1070 | 157 | 4.36 | 28 | 37 |
|  | 225 | 135 | 713 | 1070 | 157 | 4.28 | 28 | 38 |
|  | 315 | 90 | 713 | 1070 | 157 | 4.48 | 28 | 27 |
|  | 225 | 90 | 713 | 1070 | 157 | 4.67 | 28 | 43 |
|  | 450 | 0 | 713 | 1070 | 157 | 4.85 | 90 | 43 |
|  | 315 | 45 | 713 | 1070 | 157 | 4.52 | 90 | 57 |
|  | 270 | 90 | 713 | 1070 | 157 | 4.36 | 90 | 44 |
|  | 225 | 135 | 713 | 1070 | 157 | 4.28 | 90 | 43.5 |
|  | 315 | 90 | 713 | 1070 | 157 | 4.48 | 90 | 34 |
|  | 225 | 90 | 713 | 1070 | 157 | 4.67 | 90 | 47 |
|  | 450 | 0 | 713 | 1070 | 157 | 4.85 | 120 | 47 |
|  | 315 | 45 | 713 | 1070 | 157 | 4.52 | 120 | 58 |
|  | 270 | 90 | 713 | 1070 | 157 | 4.36 | 120 | 47.5 |
|  | 225 | 135 | 713 | 1070 | 157 | 4.28 | 120 | 48 |
|  | 315 | 90 | 713 | 1070 | 157 | 4.48 | 120 | 38 |
|  | 225 | 90 | 713 | 1070 | 157 | 4.67 | 120 | 47 |
| 18 | 380 | 0 | 960 | 825 | 185 | 0 | 7 | 35 |
|  | 323 | 57 | 955 | 825 | 185 | 0 | 7 | 35.5 |
|  | 266 | 114 | 947 | 825 | 185 | 0 | 7 | 33 |
|  | 209 | 171 | 940 | 825 | 185 | 0 | 7 | 31 |
|  | 152 | 228 | 933 | 825 | 185 | 0 | 7 | 28 |
|  | 380 | 57 | 907 | 825 | 185 | 0 | 7 | 48 |
|  | 380 | 0 | 960 | 825 | 185 | 0 | 28 | 47.5 |
|  | 323 | 57 | 955 | 825 | 185 | 0 | 28 | 47 |
|  | 266 | 114 | 947 | 825 | 185 | 0 | 28 | 50 |
|  | 209 | 171 | 940 | 825 | 185 | 0 | 28 | 47 |
|  | 152 | 228 | 933 | 825 | 185 | 0 | 28 | 42 |
|  | 380 | 57 | 907 | 825 | 185 | 0 | 28 | 60 |
|  | 380 | 0 | 960 | 825 | 185 | 0 | 91 | 52 |
|  | 323 | 57 | 955 | 825 | 185 | 0 | 91 | 56 |
|  | 266 | 114 | 947 | 825 | 185 | 0 | 91 | 55 |
|  | 209 | 171 | 940 | 825 | 185 | 0 | 91 | 48 |
|  | 152 | 228 | 933 | 825 | 185 | 0 | 91 | 46 |
|  | 380 | 57 | 907 | 825 | 185 | 0 | 91 | 63.5 |
| 19 | 972 | 0 | 972 | 0 | 216 | 45 | 7 | 93 |
|  | 756 | 0 | 972 | 0 | 216 | 52.5 | 7 | 78 |
|  | 756 | 216 | 972 | 0 | 216 | 41.6 | 7 | 82 |
|  | 864 | 35.6 | 972 | 0 | 216 | 43.4 | 7 | 89 |
|  | 756 | 71.3 | 972 | 0 | 216 | 46.5 | 7 | 82 |
|  | 648 | 108 | 972 | 0 | 216 | 50.8 | 7 | 77 |
|  | 972 | 0 | 972 | 0 | 216 | 45 | 28 | 118 |
|  | 756 | 0 | 972 | 0 | 216 | 52.5 | 28 | 98 |
|  | 756 | 216 | 972 | 0 | 216 | 41.6 | 28 | 111 |
|  | 864 | 35.6 | 972 | 0 | 216 | 43.4 | 28 | 118 |
|  | 756 | 71.3 | 972 | 0 | 216 | 46.5 | 28 | 122 |
|  | 648 | 108 | 972 | 0 | 216 | 50.8 | 28 | 118 |
|  | 972 | 0 | 972 | 0 | 216 | 45 | 90 | 125 |
|  | 756 | 0 | 972 | 0 | 216 | 52.5 | 90 | 109 |
|  | 756 | 216 | 972 | 0 | 216 | 41.6 | 90 | 122 |
|  | 864 | 35.6 | 972 | 0 | 216 | 43.4 | 90 | 130 |
|  | 756 | 71.3 | 972 | 0 | 216 | 46.5 | 90 | 135 |
|  | 648 | 108 | 972 | 0 | 216 | 50.8 | 90 | 128 |
| 20 | 553.6 | 0 | 795 | 810 | 221.4 | 1.1 | 28 | 39.39 |
|  | 470.6 | 64.3 | 795 | 810 | 221.4 | 1.1 | 28 | 43.96 |
|  | 415.2 | 107.2 | 795 | 810 | 221.4 | 1.1 | 28 | 42.57 |
|  | 359.8 | 150 | 795 | 810 | 221.4 | 1.1 | 28 | 35.22 |
|  | 553.6 | 0 | 397.5 | 810 | 221.4 | 1.1 | 28 | 38.29 |
|  | 470.6 | 64.3 | 397.5 | 810 | 221.4 | 1.1 | 28 | 40.85 |
|  | 415.2 | 107.2 | 397.5 | 810 | 221.4 | 1.1 | 28 | 35.77 |
|  | 359.8 | 150 | 397.5 | 810 | 221.4 | 1.1 | 28 | 34 |
|  | 553.6 | 0 | 0 | 810 | 221.4 | 1.1 | 28 | 37.7 |
|  | 470.6 | 64.3 | 0 | 810 | 221.4 | 1.1 | 28 | 39.76 |
|  | 415.2 | 107.2 | 0 | 810 | 221.4 | 1.1 | 28 | 34.17 |
|  | 359.8 | 150 | 0 | 810 | 221.4 | 1.1 | 28 | 33.24 |
|  | 332 | 150 | 0 | 810 | 221.4 | 1.1 | 28 | 33.49 |
|  | 304.5 | 150 | 0 | 810 | 221.4 | 1.1 | 28 | 35.06 |
|  | 276.8 | 150 | 0 | 810 | 221.4 | 1.1 | 28 | 32.06 |
|  | 249.1 | 150 | 0 | 810 | 221.4 | 1.1 | 28 | 29.87 |
| 21 | 665 | 0 | 553 | 1108 | 186.2 | 3 | 7 | 27.3 |
|  | 665 | 0 | 553 | 1108 | 186.2 | 3.1 | 7 | 36.6 |
|  | 665 | 0 | 553 | 1108 | 186.2 | 3.1 | 7 | 37.4 |
|  | 665 | 0 | 553 | 1108 | 186.2 | 3.2 | 7 | 39.2 |
|  | 665 | 0 | 553 | 1108 | 186.2 | 3.3 | 7 | 40.9 |
|  | 665 | 0 | 553 | 1108 | 186.2 | 3.3 | 7 | 40.4 |
|  | 665 | 20 | 442.4 | 1108 | 186.2 | 3 | 7 | 28.1 |
|  | 665 | 20 | 442.4 | 1108 | 186.2 | 3.1 | 7 | 37.9 |
|  | 665 | 20 | 442.4 | 1108 | 186.2 | 3.1 | 7 | 38.7 |
|  | 665 | 20 | 442.4 | 1108 | 186.2 | 3.2 | 7 | 40.3 |
|  | 665 | 20 | 442.4 | 1108 | 186.2 | 3.3 | 7 | 42.2 |
|  | 665 | 20 | 442.4 | 1108 | 186.2 | 3.3 | 7 | 41.9 |
|  | 665 | 25 | 414.8 | 1108 | 186.2 | 3 | 7 | 30.4 |
|  | 665 | 25 | 414.8 | 1108 | 186.2 | 3.1 | 7 | 39.3 |
|  | 665 | 25 | 414.8 | 1108 | 186.2 | 3.1 | 7 | 40.2 |
|  | 665 | 25 | 414.8 | 1108 | 186.2 | 3.2 | 7 | 43.1 |
|  | 665 | 25 | 414.8 | 1108 | 186.2 | 3.3 | 7 | 44.3 |
|  | 665 | 25 | 414.8 | 1108 | 186.2 | 3.3 | 7 | 44.1 |
|  | 665 | 30 | 387.1 | 1108 | 186.2 | 3 | 7 | 29.5 |
|  | 665 | 30 | 387.1 | 1108 | 186.2 | 3.1 | 7 | 38.7 |
|  | 665 | 30 | 387.1 | 1108 | 186.2 | 3.1 | 7 | 39.4 |
|  | 665 | 30 | 387.1 | 1108 | 186.2 | 3.2 | 7 | 41.3 |
|  | 665 | 30 | 387.1 | 1108 | 186.2 | 3.3 | 7 | 43.1 |
|  | 665 | 30 | 387.1 | 1108 | 186.2 | 3.3 | 7 | 42.9 |
|  | 665 | 0 | 553 | 1108 | 186.2 | 3 | 28 | 41 |
|  | 665 | 0 | 553 | 1108 | 186.2 | 3.1 | 28 | 55 |
|  | 665 | 0 | 553 | 1108 | 186.2 | 3.1 | 28 | 57 |
|  | 665 | 0 | 553 | 1108 | 186.2 | 3.2 | 28 | 60 |
|  | 665 | 0 | 553 | 1108 | 186.2 | 3.3 | 28 | 62.3 |
|  | 665 | 0 | 553 | 1108 | 186.2 | 3.3 | 28 | 60.8 |
|  | 665 | 20 | 442.4 | 1108 | 186.2 | 3 | 28 | 44.7 |
|  | 665 | 20 | 442.4 | 1108 | 186.2 | 3.1 | 28 | 58.3 |
|  | 665 | 20 | 442.4 | 1108 | 186.2 | 3.1 | 28 | 59.3 |
|  | 665 | 20 | 442.4 | 1108 | 186.2 | 3.2 | 28 | 62.7 |
|  | 665 | 20 | 442.4 | 1108 | 186.2 | 3.3 | 28 | 64.7 |
|  | 665 | 20 | 442.4 | 1108 | 186.2 | 3.3 | 28 | 63.1 |
|  | 665 | 25 | 414.8 | 1108 | 186.2 | 3 | 28 | 47.2 |
|  | 665 | 25 | 414.8 | 1108 | 186.2 | 3.1 | 28 | 62.4 |
|  | 665 | 25 | 414.8 | 1108 | 186.2 | 3.1 | 28 | 61.5 |
|  | 665 | 25 | 414.8 | 1108 | 186.2 | 3.2 | 28 | 66.1 |
|  | 665 | 25 | 414.8 | 1108 | 186.2 | 3.3 | 28 | 67.9 |
|  | 665 | 25 | 414.8 | 1108 | 186.2 | 3.3 | 28 | 67.6 |
|  | 665 | 30 | 387.1 | 1108 | 186.2 | 3 | 28 | 46.3 |
|  | 665 | 30 | 387.1 | 1108 | 186.2 | 3.1 | 28 | 61.1 |
|  | 665 | 30 | 387.1 | 1108 | 186.2 | 3.1 | 28 | 60.1 |
|  | 665 | 30 | 387.1 | 1108 | 186.2 | 3.2 | 28 | 64.9 |
|  | 665 | 30 | 387.1 | 1108 | 186.2 | 3.3 | 28 | 66.2 |
|  | 665 | 30 | 387.1 | 1108 | 186.2 | 3.3 | 28 | 65.7 |
|  | 665 | 0 | 553 | 1108 | 186.2 | 3 | 90 | 49 |
|  | 665 | 0 | 553 | 1108 | 186.2 | 3.1 | 90 | 65.8 |
|  | 665 | 0 | 553 | 1108 | 186.2 | 3.1 | 90 | 69.1 |
|  | 665 | 0 | 553 | 1108 | 186.2 | 3.2 | 90 | 72.4 |
|  | 665 | 0 | 553 | 1108 | 186.2 | 3.3 | 90 | 75.1 |
|  | 665 | 0 | 553 | 1108 | 186.2 | 3.3 | 90 | 73.1 |
|  | 665 | 20 | 442.4 | 1108 | 186.2 | 3 | 90 | 53.7 |
|  | 665 | 20 | 442.4 | 1108 | 186.2 | 3.1 | 90 | 68.8 |
|  | 665 | 20 | 442.4 | 1108 | 186.2 | 3.1 | 90 | 72.8 |
|  | 665 | 20 | 442.4 | 1108 | 186.2 | 3.2 | 90 | 74.6 |
|  | 665 | 20 | 442.4 | 1108 | 186.2 | 3.3 | 90 | 78.2 |
|  | 665 | 20 | 442.4 | 1108 | 186.2 | 3.3 | 90 | 75.9 |
|  | 665 | 25 | 414.8 | 1108 | 186.2 | 3 | 90 | 58.9 |
|  | 665 | 25 | 414.8 | 1108 | 186.2 | 3.1 | 90 | 73.4 |
|  | 665 | 25 | 414.8 | 1108 | 186.2 | 3.1 | 90 | 76.4 |
|  | 665 | 25 | 414.8 | 1108 | 186.2 | 3.2 | 90 | 79.9 |
|  | 665 | 25 | 414.8 | 1108 | 186.2 | 3.3 | 90 | 82.7 |
|  | 665 | 25 | 414.8 | 1108 | 186.2 | 3.3 | 90 | 80.4 |
|  | 665 | 30 | 387.1 | 1108 | 186.2 | 3 | 90 | 57.1 |
|  | 665 | 30 | 387.1 | 1108 | 186.2 | 3.1 | 90 | 71.2 |
|  | 665 | 30 | 387.1 | 1108 | 186.2 | 3.1 | 90 | 74.1 |
|  | 665 | 30 | 387.1 | 1108 | 186.2 | 3.2 | 90 | 64.9 |
|  | 665 | 30 | 387.1 | 1108 | 186.2 | 3.3 | 90 | 66.2 |
|  | 665 | 30 | 387.1 | 1108 | 186.2 | 3.3 | 90 | 65.7 |
| 22 | 443.5 | 0 | 580 | 875 | 168.5 | 0 | 3 | 22 |
|  | 354.8 | 88.7 | 580 | 875 | 168.5 | 0 | 3 | 18 |
|  | 443.5 | 0 | 580 | 875 | 204 | 0 | 3 | 17 |
|  | 354.8 | 88.7 | 580 | 875 | 204 | 0 | 3 | 14 |
|  | 443.5 | 0 | 580 | 875 | 168.5 | 0 | 27 | 28 |
|  | 354.8 | 88.7 | 580 | 875 | 168.5 | 0 | 27 | 24 |
|  | 443.5 | 0 | 580 | 875 | 204 | 0 | 27 | 22 |
|  | 354.8 | 88.7 | 580 | 875 | 204 | 0 | 27 | 17.5 |
|  | 443.5 | 0 | 580 | 875 | 168.5 | 0 | 28 | 41 |
|  | 354.8 | 88.7 | 580 | 875 | 168.5 | 0 | 28 | 38 |
|  | 443.5 | 0 | 580 | 875 | 204 | 0 | 28 | 35 |
|  | 354.8 | 88.7 | 580 | 875 | 204 | 0 | 28 | 33 |
|  | 443.5 | 0 | 580 | 875 | 168.5 | 0 | 90 | 52 |
|  | 354.8 | 88.7 | 580 | 875 | 168.5 | 0 | 90 | 54 |
|  | 443.5 | 0 | 580 | 875 | 204 | 0 | 90 | 42 |
|  | 354.8 | 88.7 | 580 | 875 | 204 | 0 | 90 | 45 |
|  | 443.5 | 0 | 580 | 875 | 168.5 | 0 | 156 | 54 |
|  | 354.8 | 88.7 | 580 | 875 | 168.5 | 0 | 156 | 57 |
|  | 443.5 | 0 | 580 | 875 | 204 | 0 | 156 | 45 |
|  | 354.8 | 88.7 | 580 | 875 | 204 | 0 | 156 | 48 |
|  | 443.5 | 0 | 580 | 875 | 168.5 | 0 | 300 | 58.5 |
|  | 354.8 | 88.7 | 580 | 875 | 168.5 | 0 | 300 | 64 |
|  | 443.5 | 0 | 580 | 875 | 204 | 0 | 300 | 48 |
|  | 354.8 | 88.7 | 580 | 875 | 204 | 0 | 300 | 52 |
| 23 | 450 | 0 | 706 | 1059 | 157.5 | 0 | 7 | 36.68 |
|  | 427.5 | 22.5 | 704 | 1056 | 157.5 | 0 | 7 | 35.59 |
|  | 405 | 45 | 703 | 1054 | 157.5 | 0 | 7 | 39.08 |
|  | 382.5 | 67.5 | 702 | 1052 | 157.5 | 0 | 7 | 36.23 |
|  | 360 | 90 | 700 | 1050 | 157.5 | 0 | 7 | 33.09 |
|  | 337.5 | 112.5 | 698 | 1048 | 157.5 | 0 | 7 | 33 |
|  | 450 | 22.5 | 697 | 1045 | 157.5 | 0 | 7 | 38.6 |
|  | 450 | 45 | 688 | 1032 | 157.5 | 0 | 7 | 39.2 |
|  | 450 | 67.5 | 679 | 1019 | 157.5 | 0 | 7 | 40.7 |
|  | 450 | 90 | 670 | 1005 | 157.5 | 0 | 7 | 41.8 |
|  | 450 | 112.5 | 661 | 992 | 157.5 | 0 | 7 | 40.9 |
|  | 450 | 0 | 706 | 1059 | 157.5 | 0 | 28 | 44.32 |
|  | 427.5 | 22.5 | 704 | 1056 | 157.5 | 0 | 28 | 45.42 |
|  | 405 | 45 | 703 | 1054 | 157.5 | 0 | 28 | 46.45 |
|  | 382.5 | 67.5 | 702 | 1052 | 157.5 | 0 | 28 | 40.44 |
|  | 360 | 90 | 700 | 1050 | 157.5 | 0 | 28 | 39.39 |
|  | 337.5 | 112.5 | 698 | 1048 | 157.5 | 0 | 28 | 38.08 |
|  | 450 | 22.5 | 697 | 1045 | 157.5 | 0 | 28 | 45.16 |
|  | 450 | 45 | 688 | 1032 | 157.5 | 0 | 28 | 46.84 |
|  | 450 | 67.5 | 679 | 1019 | 157.5 | 0 | 28 | 47.5 |
|  | 450 | 90 | 670 | 1005 | 157.5 | 0 | 28 | 49.2 |
|  | 450 | 112.5 | 661 | 992 | 157.5 | 0 | 28 | 48.43 |
|  | 450 | 0 | 706 | 1059 | 157.5 | 0 | 56 | 45.51 |
|  | 427.5 | 22.5 | 704 | 1056 | 157.5 | 0 | 56 | 48.08 |
|  | 405 | 45 | 703 | 1054 | 157.5 | 0 | 56 | 49.18 |
|  | 382.5 | 67.5 | 702 | 1052 | 157.5 | 0 | 56 | 43.15 |
|  | 360 | 90 | 700 | 1050 | 157.5 | 0 | 56 | 41.12 |
|  | 337.5 | 112.5 | 698 | 1048 | 157.5 | 0 | 56 | 39.73 |
|  | 450 | 22.5 | 697 | 1045 | 157.5 | 0 | 56 | 47.2 |
|  | 450 | 45 | 688 | 1032 | 157.5 | 0 | 56 | 48.76 |
|  | 450 | 67.5 | 679 | 1019 | 157.5 | 0 | 56 | 50.9 |
|  | 450 | 90 | 670 | 1005 | 157.5 | 0 | 56 | 54.84 |
|  | 450 | 112.5 | 661 | 992 | 157.5 | 0 | 56 | 52.7 |
| 24 | 425 | 0 | 680 | 1100 | 212.5 | 1.4 | 7 | 28 |
|  | 340 | 95.2 | 584.8 | 1100 | 212.5 | 1.45 | 7 | 28.5 |
|  | 340 | 102 | 578 | 1100 | 212.5 | 1.5 | 7 | 29 |
|  | 340 | 108.8 | 571 | 1100 | 212.5 | 1.55 | 7 | 30 |
|  | 340 | 115.6 | 654.4 | 1100 | 212.5 | 1.65 | 7 | 29 |
|  | 340 | 122.4 | 557.6 | 1100 | 212.5 | 1.7 | 7 | 27 |
|  | 340 | 129.2 | 550.8 | 1100 | 212.5 | 1.8 | 7 | 24 |
|  | 340 | 136 | 544 | 1100 | 212.5 | 1.9 | 7 | 22.5 |
|  | 425 | 0 | 680 | 1100 | 212.5 | 1.4 | 28 | 33 |
|  | 340 | 95.2 | 584.8 | 1100 | 212.5 | 1.45 | 28 | 35.5 |
|  | 340 | 102 | 578 | 1100 | 212.5 | 1.5 | 28 | 37 |
|  | 340 | 108.8 | 571 | 1100 | 212.5 | 1.55 | 28 | 38.5 |
|  | 340 | 115.6 | 654.4 | 1100 | 212.5 | 1.65 | 28 | 37 |
|  | 340 | 122.4 | 557.6 | 1100 | 212.5 | 1.7 | 28 | 34 |
|  | 340 | 129.2 | 550.8 | 1100 | 212.5 | 1.8 | 28 | 32 |
|  | 340 | 136 | 544 | 1100 | 212.5 | 1.9 | 28 | 29.3 |
|  | 425 | 0 | 680 | 1100 | 212.5 | 1.4 | 90 | 41 |
|  | 340 | 95.2 | 584.8 | 1100 | 212.5 | 1.45 | 90 | 43 |
|  | 340 | 102 | 578 | 1100 | 212.5 | 1.5 | 90 | 45 |
|  | 340 | 108.8 | 571 | 1100 | 212.5 | 1.55 | 90 | 49 |
|  | 340 | 115.6 | 654.4 | 1100 | 212.5 | 1.65 | 90 | 48 |
|  | 340 | 122.4 | 557.6 | 1100 | 212.5 | 1.7 | 90 | 47 |
|  | 340 | 129.2 | 550.8 | 1100 | 212.5 | 1.8 | 90 | 46 |
|  | 340 | 136 | 544 | 1100 | 212.5 | 1.9 | 90 | 35 |
| 25 | 449 | 0 | 500 | 0 | 226 | 38 | 7 | 68.9 |
|  | 421 | 125 | 500 | 0 | 212 | 36 | 7 | 91.8 |
|  | 393 | 250 | 500 | 0 | 197 | 34 | 7 | 102.2 |
|  | 365 | 375 | 500 | 0 | 183 | 31 | 7 | 89.8 |
|  | 449 | 0 | 450 | 0 | 226 | 38 | 7 | 78.9 |
|  | 421 | 125 | 450 | 0 | 212 | 36 | 7 | 106.9 |
|  | 393 | 250 | 450 | 0 | 197 | 34 | 7 | 122.2 |
|  | 365 | 375 | 450 | 0 | 183 | 31 | 7 | 105 |
|  | 449 | 0 | 500 | 0 | 226 | 38 | 28 | 81.2 |
|  | 421 | 125 | 500 | 0 | 212 | 36 | 28 | 109.6 |
|  | 393 | 250 | 500 | 0 | 197 | 34 | 28 | 124.2 |
|  | 365 | 375 | 500 | 0 | 183 | 31 | 28 | 109.9 |
|  | 449 | 0 | 450 | 0 | 226 | 38 | 28 | 92.1 |
|  | 421 | 125 | 450 | 0 | 212 | 36 | 28 | 132.5 |
|  | 393 | 250 | 450 | 0 | 197 | 34 | 28 | 154.2 |
|  | 365 | 375 | 450 | 0 | 183 | 31 | 28 | 135.6 |
| 26 | 442 | 78 | 665.6 | 1144 | 130 | 10.3 | 7 | 78 |
|  | 468 | 52 | 665.6 | 1144 | 182.8 | 2.29 | 7 | 54 |
|  | 494 | 26 | 665.6 | 1144 | 234 | 0.57 | 7 | 43 |
|  | 442 | 78 | 665.6 | 1144 | 130 | 10.3 | 28 | 88 |
|  | 468 | 52 | 665.6 | 1144 | 182.8 | 2.29 | 28 | 63 |
|  | 494 | 26 | 665.6 | 1144 | 234 | 0.57 | 28 | 55 |
